# Ursodeoxycholic acid alleviates high-fat diet-induced liver injury by modulating gut microbiota-mediated bile acid metabolism: an integrated microbiota-metabolomics analysis

**DOI:** 10.3389/fnut.2026.1714100

**Published:** 2026-01-26

**Authors:** Xueyun Dong, Wen Sun, Hao Xu, Yunhan Xie, Jiayuan He, Xuehui Liu, Xinyu Liu, Asmaa Ali, Min Chen, Leilei Zhang, Liang Wu, Keke Shao

**Affiliations:** 1 Department of Laboratory Medicine, School of Medicine, Jiangsu University, Zhenjiang, China; 2 Department of Laboratory Medicine, Yancheng First Hospital Affiliated of Nanjing University Medical School, The Yancheng Clinical College of Xuzhou Medical University, The First People’s Hospital of Yancheng, Yancheng, China; 3 Critical Care Medicine, Jurong Hospital Affiliated to Jiangsu University, Zhenjiang, China; 4 Health Testing Center, Zhenjiang Center for Disease Control and Prevention, Zhenjiang, China; 5 Department of Pulmonary Medicine, Abbassia Chest Hospital, EMOH, Cairo, Egypt; 6 Public Experiment and Service Center, Jiangsu University, Zhenjiang, China; 7 Molecular Medical Research Center, Yancheng Clinical Medical College of Jiangsu University, Yancheng, China

**Keywords:** bile acid metabolism, gut microbiota, gut-liver axis, non-alcoholic fatty liver disease, ursodeoxycholic acid

## Abstract

**Purpose:**

Ursodeoxycholic acid (UDCA), a naturally occurring bile acid with established hepatoprotective properties, has garnered attention for its potential role in metabolic health. This study provides scientific validation for these traditional uses by demonstrating UDCA’s protective mechanisms against non-alcoholic fatty liver disease (NAFLD) through gut microbiota modulation and metabolic regulation. This study elucidates the therapeutic mechanisms of UDCA against high-fat diet-induced NAFLD through integrated microbiota-metabolomics analysis.

**Methods:**

Using a 12-week murine NAFLD model, oral UDCA (15 mg/kg/day and 30 mg/kg/day) was administered to evaluate its hepatoprotective effects. Hepatic steatosis and injury were assessed via serum ALT/AST levels, lipid profiles, and histopathology. Ultra-performance liquid chromatography–tandem mass spectrometry (UPLC-MS/MS) quantified bile acid metabolites, while 16S rRNA sequencing analyzed gut microbiota composition. Serum metabolomics and network pharmacology were employed to identify metabolic pathways and mechanistic targets, respectively. Molecular analyses (qPCR/Western blot) assessed PPARγ/Nrf2/NF-κB signaling.

**Results:**

UDCA treatment significantly ameliorated high-fat diet-induced NAFLD, as demonstrated by improved serum ALT/AST levels, attenuated hepatic steatosis, and reduced histopathological damage. UPLC-MS/MS analysis revealed a marked reorganization of bile acid metabolism, characterized by elevated non-12α-hydroxylated bile acids (UDCA, TUDCA) and enhanced alternative synthesis *via* CYP27A1 upregulation. 16S rRNA sequencing identified UDCA-driven restructuring of the gut microbiota, with specific enrichment of short-chain fatty acid-producing *Muribaculum* spp. and suppression of pro-inflammatory *Prevotella* (*CAG-485*). Serum metabolomics further confirmed these benefits, showing increased eicosapentaenoic acid (anti-inflammatory) and decreased long-chain acylcarnitines (lipid peroxidation markers). At the molecular level, UDCA activated PPARγ/Nrf2 antioxidative signaling while inhibiting NF-κB-mediated inflammation, and network pharmacology analysis identified 225 potential targets (including TNF-*α*, IL6, and NF-κB) within lipid/atherosclerosis pathways, collectively underscoring UDCA’s multimodal protective mechanisms against NAFLD.

**Conclusion:**

These findings validate UDCA’s multifaceted hepatoprotection *via* microbiota-bile acid crosstalk and metabolic-inflammatory modulation. The study provides a mechanistic basis for UDCA’s traditional use in hepatobiliary disorders by integrating microbial, metabolic, and molecular evidence.

## Introduction

Non-alcoholic fatty liver disease (NAFLD) encompasses a spectrum of hepatic pathologies ranging from simple steatosis to progressive hepatocyte injury and eventual fibrosis, representing the predominant cause of chronic liver disease globally. Epidemiological data reveal a prevalence of 29.1% in the general population, with risk factors including genetic predisposition, obesity, insulin resistance, and gut dysbiosis (1). Despite extensive research, the intricate pathogenesis and heterogeneous nature of NAFLD have thus far precluded the approval of any pharmacological therapy for this condition (2).

The development and progression of NAFLD follows a “two-hit” pathogenesis, fundamentally characterized by chronic inflammatory responses and metabolic disturbances leading to hepatic injury (3). The first hit involves high-calorie diet-induced dyslipidemia, causing aberrant intrahepatic accumulation of free fatty acids and triglycerides, resulting in simple steatosis (4). This process is exacerbated by malonyl-CoA accumulation, which inhibits carnitine palmitoyltransferase-1 (CPT-1) activity, impairing fatty acid *β*-oxidation, while lipotoxicity simultaneously promotes reactive oxygen species (ROS) overproduction, triggering mitochondrial dysfunction and endoplasmic reticulum stress (5, 6). The second hit, driven by oxidative stress-mediated chronic inflammation, represents the critical transition to non-alcoholic steatohepatitis (NASH) and fibrosis (7, 8). Here, dysregulation of the Nrf2/PPARγ antioxidant axis leads to hyperactivation of NF-κB signaling, increasing pro-inflammatory cytokine release (e.g., TNF-*α*, IL-1β) and establishing a vicious “oxidative stress-inflammation-fibrosis” cascade (9, 10). Emerging evidence highlights gut dysbiosis as a key amplifier of this cycle: (1) bacterial endotoxin (LPS) translocates into circulation, activating TLR4/MyD88 pathways to exacerbate hepatic inflammation (11); and (2) Microbiota-dependent bile acid metabolism disruptions (e.g., suppression of farnesoid X receptor [FXR] and G protein-coupled bile acid receptor 5 [TGR5] signaling) further compromise hepatic lipid clearance (12, 13). This bidirectional gut-liver interplay underscores NAFLD’s complex, self-perpetuating pathology (14).

Bile acids (BAs), essential microbial metabolites derived from the gut microbiota, serve as central regulators of host nutrient absorption, metabolic homeostasis, and immune balance (15–17). Hepatic synthesis of primary BAs [e.g., cholic acid (CA), chenodeoxycholic acid (CDCA)] occurs *via* a multistep enzymatic cascade involving cytochrome P450 family 7 subfamily A member 1 (CYP7A1), cytochrome P450 family 8 subfamily B member 1 (CYP8B1), cytochrome P450 family 27 subfamily A member 1 (CYP27A1), and cytochrome P450 family 7 subfamily B member 1 (CYP7B1), followed by intestinal secretion where gut microbiota metabolize them through deconjugation and 7α-dehydroxylation to generate secondary BAs [e.g., deoxycholic acid (DCA), lithocholic acid (LCA)] with enhanced signaling potency (18). Approximately 95% of BAs undergo enterolepatic recirculation through apical sodium-dependent bile acid transporter (ASBT)-mediated ileal reabsorption, while the non-absorbed fraction acts *via* the microbiota-BA-host receptor axis to systemically modulate metabolic homeostasis (19, 20). These BAs enter systemic circulation to activate nuclear receptors (FXR, PXR, VDR) and membrane receptors (TGR5, S1PR2), thereby governing glucose/lipid metabolism, energy expenditure, and inflammatory responses (21, 22). Critically, microbiota-dependent BA remodeling (e.g., by *Clostridium scindens* and *Bacteroides* spp.) drives metabolic disorders like obesity, diabetes, and NAFLD by reshaping BA pools and altering FXR/TGR5 signaling (23). Thus, BAs transcend their classical role as digestive surfactants, emerging as pivotal “gut-liver-peripheral organ” messengers whose dysregulation underpins metabolic syndrome, inflammatory bowel disease, and hepatic pathogenesis.

Ursodeoxycholic acid (UDCA), a secondary bile acid with well-documented clinical applications, traces its medicinal origins to traditional Chinese therapeutics, notably bear bile (e.g., *Ursus thibetanus* bile) used historically for hepatobiliary disorders (24, 25). Modern research has not only validated UDCA’s efficacy in mitigating hepatic inflammation and fibrosis but also established it as the first FDA-approved therapy for primary biliary cholangitis (PBC) (8, 26). Its pleiotropic mechanisms involve: (1) suppression of hepatic farnesoid X receptor (FXR)-small heterodimer partner (SHP) signaling, reducing bile acid synthesis and cholestatic liver injury (27); and (2) activation of intestinal FXR-fibroblast growth factor 15/19 (FGF15/19) axis, which feedback-inhibits bile acid production while enhancing enterohepatic circulation (28, 29). Clinical and preclinical studies further demonstrate UDCA’s capacity to remodel the hepatic inflammatory milieu and counteract fibrogenesis via inhibition of hepatic stellate cell activation and collagen deposition, highlighting its therapeutic potential in NASH and other chronic liver diseases (30, 31). These findings not only bridge traditional medicine with modern pharmacology but also underscore the synergistic potential of integrating Eastern and Western approaches in hepatology (32).

This study systematically elucidates UDCA’s mechanisms in NAFLD through a gut microbiota-bile acid axis lens—an approach distinct from prior cholestasis-focused research. While Zhang et al. (31) employed similar UPLC-MS/MS bile acid analysis, our integration with network pharmacology and in silico validation creates unprecedented predictive power. Notably, our ICR mouse model recapitulates NAFLD microbiota dysbiosis, enhancing translational relevance. The concurrent profiling of microbial taxa (e.g., Bacteroides UDCA-metabolizing clusters) and host signaling pathways represents a significant methodological advancement over previous fragmentary analyses.

## Materials and methods

### Animals and experimental design

Six-week-old male ICR mice (25 ± 4 g) of specific pathogen-free (SPF) grade were obtained from Jiangsu Wukong Biotechnology Co., Ltd. and housed at the Animal Experiment Center of Jiangsu University under controlled conditions (temperature 23 ± 2 °C, relative humidity 60 ± 5%, 12-h light/dark cycle). Forty mice were randomly allocated into four groups (*n* = 10/group): (1) normal control (NC) group fed with standard chow (XM001, Xietong Co., Nanjing, China); (2) non-alcoholic fatty liver disease (NAFLD) model group; (3) high ursodeoxycholic acid (UDCA)-treated group (UDCAH, 30 mg/kg/day); and (4) low UDCA-treated group (UDCAL, 15 mg/kg/day), both receiving 60% high-fat diet (HFM001, Xietong Co.) for 12 weeks. From week 4 onward, the UDCAL and UDCAH groups received daily oral gavage of UDCA (15 mg/kg and 30 mg/kg; U5127, Sigma-Aldrich) dissolved in saline, while NC and NAFLD groups received equivalent volumes (200 μL) of saline. Mouse anesthesia and euthanasia procedures were performed using the Shenzhen RWD small animal anesthesia system (Shenzhen, China). Induction anesthesia was achieved with 3% sevoflurane (Sichuan Kelun Pharmaceutical Co., Ltd., China; National Drug Approval No. H20183032) delivered at an oxygen flow rate of 1 L/min. Once deep anesthesia was confirmed (loss of righting reflex and absence of toe-pinch response), cervical dislocation was rapidly performed for euthanasia. Upon operations completion, serum, colonic contents, and liver tissues were collected for subsequent analyses.

### Cell culture and experimental treatments

Human hepatocellular carcinoma HepG2 cells (ATCC^®^ HB-8065™) were sourced from Wuhan Procell Life Science and Technology Co., Ltd. (Catalog #: CL-0103). Cell authentication was performed through short tandem repeat (STR) profiling, and routine mycoplasma contamination testing was conducted to ensure cell line integrity. The cells were maintained in RPMI 1640 medium (WISENT, Nanjing, China) containing 10% fetal bovine serum (FBS; WISENT) at 37 °C in a humidified 5% CO₂ atmosphere.

Oleic acid (OA; Sigma-Aldrich) was dissolved in dimethyl sulfoxide (DMSO) to prepare a 200 mM stock solution. At 80% confluence, HepG2 cells were synchronized in serum-free medium for 4 h prior to treatment with 0.2 mM OA to establish the lipid overload model. Ursodeoxycholic acid (UDCA) was dissolved in DMSO to yield a 100 mg/mL stock solution, which was subsequently diluted in culture medium to achieve a final concentration of 100 μM. For experimental groups, cells were co-treated with OA (0.2 mM) and UDCA (100 μM) for 24 h. Vehicle control cells received equivalent volumes of DMSO (0.2% final concentration), maintaining consistency with solvent exposure across all treatment groups.

### Network pharmacology and molecular docking analysis of the mechanism of UDCA in treating NAFLD

To predict the potential targets of UDCA in the treatment of NAFLD, we first conducted target prediction using multiple databases. The SMILES format of UDCA was retrieved from PubChem and submitted to the PharmMapper and SwissTarget Prediction databases to generate potential target profiles. Additionally, the Comparative Toxicogenomics Database (CTD) was queried for UDCA-related targets by searching its chemical structure. The predicted targets from all three databases were combined to establish a comprehensive UDCA target dataset. Meanwhile, NAFLD-related genes were retrieved from the Therapeutic Target Database (TTD), GeneCards, and the Online Mendelian Inheritance in Man (OMIM) database using “non-alcoholic fatty liver disease” as the search term. A Venn diagram was constructed to identify the overlapping targets between UDCA and NAFLD.

The intersecting targets were subsequently imported into the STRING database to construct a protein–protein interaction (PPI) network, which was then analyzed using Cytoscape 3.9.0. The CytoHubba plugin was employed to screen for hub genes based on degree centrality. Gene Ontology (GO) enrichment analysis (including biological processes, molecular functions, and cellular components) and Kyoto Encyclopedia of Genes and Genomes (KEGG) pathway analysis were performed using the DAVID database (with a significance threshold of *p* < 0.05), and the results were visualized using RStudio.

To further validate the network pharmacology findings, molecular docking analysis was conducted. The 3D structure of UDCA was obtained from PubChem and converted using OpenBabel 2.4.1. The predicted full-length structure of the P65 protein (UniProtKB ID: AF-Q04206-F1) was retrieved, and water molecules and ligands were removed using PyMOL. AutoDockTools-1.5.7 was then utilized to prepare the ligand (adding hydrogens, removing water, and computing charges) before performing molecular docking. The binding affinity (kcal/mol) was calculated, and the resulting interactions were visualized in PyMOL.

### Analysis of serum biochemical parameters and hepatic inflammatory factors

Serum lipid profiles including total cholesterol (TC, A111-1), triglycerides (TG, A110-1), low-density lipoprotein cholesterol (LDL-C, A113-1), and high-density lipoprotein cholesterol (HDL-C, A112-1) were determined using commercial assay kits (Nanjing Jiancheng Bioengineering Institute, China) according to the manufacturer’s instructions with modifications from Sun et al. Blood samples were centrifuged at 3,000 × g for 15 min at 4 °C to obtain serum, which was immediately analyzed or stored at −80 °C. For qPCR analysis, total RNA was extracted from liver tissue and reverse transcribed using HiScript III RT SuperMix (R323-01, Vazyme, Nanjing, China), followed by real-time PCR with ChamQ Universal SYBR qPCR Master Mix (Q711-02, Vazyme) on a QuantStudio 3 system (Applied Biosystems). The PCR protocol consisted of initial denaturation at 95 °C for 30 s, followed by 40 cycles of 95 °C for 5 s and 60 °C for 30 s. Gene expression levels of inflammatory factors were normalized to *β*-actin and calculated using the 2^–ΔΔCt^ method, with primer sequences provided in Supplementary Table S1. All measurements were performed in technical triplicates.

### Western blotting analysis

Total proteins were extracted from fresh tissues using RIPA lysis buffer, denatured, and separated by SDS-PAGE before electrophoretic transfer to PVDF membranes. The membranes were blocked with 5% non-fat milk for 1 h at room temperature and subsequently incubated overnight at 4 °C with primary antibodies obtained from Proteintech (Wuhan, China). After washing with TBST (3 × 10 min), the membranes were incubated with corresponding HRP-conjugated secondary antibodies (Proteintech) for 1 h at room temperature. Protein bands were visualized using enhanced chemiluminescence (ECL) with an e-blot imaging system, and quantification was performed using ImageJ software (NIH, United States).

### Histopathological and lipid deposition analysis

Liver tissues (approximately 2 mm^3^) were fixed in 4% paraformaldehyde for at least 16 h, paraffin-embedded, and sectioned at 4 μm thickness for histological examination. Hematoxylin–eosin (H&E) staining was performed by the Department of Pathology at Yancheng First People’s Hospital to evaluate inflammatory damage, while cryosections were stained with Oil Red O (Sigma-Aldrich, O1391) to assess lipid deposition using standard protocols. Microscopic examination was conducted to evaluate hepatic steatosis (scored 0–3), lobular inflammation (0–3), and hepatocellular ballooning (0–2), with the NAFLD activity score (NAS) calculated as the sum of these individual scores. Two independent pathologists blinded to the experimental groups performed all histological assessments to ensure objective evaluation.

### Gut microbiota 16S rRNA gene sequencing and bioinformatic analysis

Colonic contents from mice were immediately flash-frozen in liquid nitrogen and stored at −80 °C until DNA extraction. Genomic DNA was extracted using the QIAamp Fast DNA Stool Mini Kit (Germany) following the manufacturer’s protocol. The V3–V4 hypervariable regions of the bacterial 16S rRNA gene were amplified using primers [341F (5′-CCTACGGGNGGCWGCAG-3′) and 805R (5′-GACTACHVGGGTATCTAATCC-3′)]. PCR products were purified, quantified, and pooled in equimolar ratios. Paired-end sequencing was performed on the Illumina platform (NovaSeq 6,000) by Wekemo Tech Group Co., Ltd. (Shenzhen, China).

Raw sequences were demultiplexed and quality-filtered to remove adapters, low-quality reads (Q-score <20), and chimeras. High-quality reads were clustered into operational taxonomic units (OTUs). Community diversity metrics (*α*-diversity: Shannon, Chao1; *β*-diversity: weighted/unweighted UniFrac) were calculated with QIIME2. Principal component analysis (PCA), non-metric multidimensional scaling (NMDS), and hierarchical clustering were performed using R packages to evaluate microbial shifts and treatment effects.

### Serum bile acid profiling and metabolomic analysis

Targeted Bile Acid Analysis: Fresh serum samples were thawed on ice and proteins were precipitated by adding 4 volumes of ice-cold methanol containing internal standards (d4-glycocholic acid). After vortexing (30 s) and centrifugation (15,000 × g, 10 min, 4 °C), supernatants were analyzed using ultra-performance liquid chromatography–tandem mass spectrometry (UPLC-MS/MS; Waters Acquity I-Class/Xevo TQ-S, United States) at Zhenjiang Center for Disease Control and Prevention. Separation was achieved on a C18 column (Waters Acquity UPLC BEH C18, 2.1 × 100 mm, 1.7 μm) with mobile phase A (0.1% formic acid in water) and B (0.1% formic acid in acetonitrile) at 40 °C. The gradient elution program was: 0–2 min 10% B, 2–8 min 10–90% B, 8–9 min 90% B, 9–9.1 min 90–10% B, 9.1–11 min 10% B at 0.4 mL/min flow rate. MS/MS detection employed electrospray ionization in negative mode with optimized parameters. Quantification used external calibration curves (1–1,000 ng/mL, *R*^2^ > 0.99).

Serum samples (50 μL) were extracted with 200 μL ice-cold methanol/acetonitrile/water (2:2:1) containing isotopically labeled internal standards. After vortexing (60 s), ultrasonication (10 min, ice bath), and centrifugation (14,000 × g, 20 min, 4 °C), supernatants were analyzed by UPLC-quadrupole time-of-flight-MS/MS (Agilent 1,290 Infinity LC/6550 Q-TOF) with electrospray ionization (±) modes (details: column—Agilent ZORBAX Eclipse Plus C18 RRHD, 2.1 × 100 mm, 1.8 μm; flow rate 0.3 mL/min; gradient 0–14 min 5–95% B). Mass spectra were acquired in full scan mode (m/z 50–1,000) with auto MS/MS fragmentation (collision energy 20–40 eV).

Raw UPLC-MS/MS data were processed using Progenesis QI for peak picking, alignment, and normalization (to total ion current/internal standards). Multivariate analyses including PCA and OPLS-DA were performed in metaX software with unit variance scaling. Metabolites were identified by matching accurate mass (<10 ppm) and MS/MS spectra against HMDB^1^ and Metlin^2^ databases. Differential metabolites were filtered by VIP > 1.0 (from OPLS-DA) and *p* < 0.05 (two-tailed Student’s *t*-test with FDR correction). Pathway analysis used KEGG Mapper^3^ with significance thresholds (*p* < 0.05, impact > 0.1).

### Statistical analysis

All data were analyzed using SPSS 20.0 software (SPSS Inc., Chicago, IL, United States) and expressed as mean ± standard deviation (SD). Group comparisons were performed using Student’s *t*-test or one-way analysis of variance (ANOVA) followed by appropriate *post-hoc* tests, with *p* < 0.05 considered statistically significant. Data visualization was conducted using GraphPad Prism software (GraphPad Software, San Diego, CA, United States) and the Bioincloud platform.^4^

## Results

### UDCA ameliorates hepatic injury and steatosis in NAFLD mice

**Figure 1:**
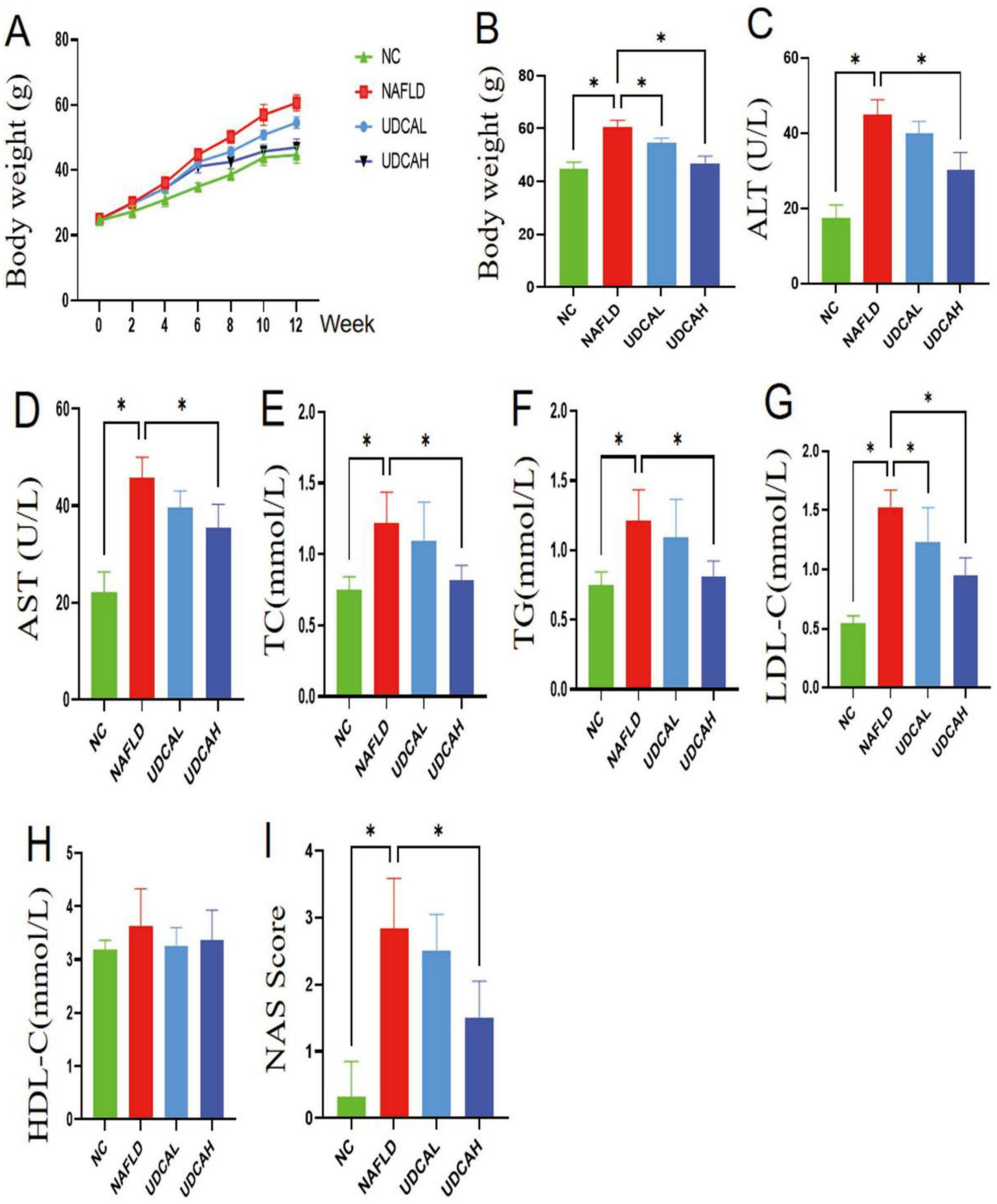
Effects of high and low concentration UDCA on body weight and serum biochemical parameters in experimental mice (*n* = 6). **(A)** Dynamic changes in body weight. **(B)** Terminal body weight measured during the final week. **(C)** Serum alanine aminotransferase (ALT). **(D)** Serum aspartate aminotransferase (AST). **(E)** Serum total cholesterol (TC). **(F)** Serum triglycerides (TG). **(G)** Serum high-density lipoprotein cholesterol (HDL-C). **(H)** Serum low-density lipoprotein cholesterol (LDL-C). **(I)** NAFLD activity score (NAS). Values are presented as mean ± SEM. ^*^p < 0.05 indicates statistically significant differences versus control group.

During the experimental period, mice fed a high-fat diet (HFD) exhibited rapid weight gain, which was significantly attenuated by high concentration UDCA treatment (UDCAH group) (*p* < 0.05). At the endpoint, UDCA-administered mice displayed markedly lower body weight compared to the NAFLD group. Importantly, serum levels of hepatic injury markers (ALT and AST) were substantially elevated in NAFLD mice but significantly reduced by high concentration UDCA intervention (*p* < 0.05) (Figure 1), demonstrating its hepatoprotective effect against steatosis-induced damage. Moreover, UDCA effectively counteracted HFD-induced dyslipidemia, as evidenced by significantly lower serum concentrations of TC, TG, and LDL in the treatment group compared to untreated NAFLD controls (*p* < 0.05) (Figure 1). These findings collectively indicate that UDCA not only mitigates hepatic steatosis and injury but also restores metabolic homeostasis in NAFLD progression.

**Figure 2:**
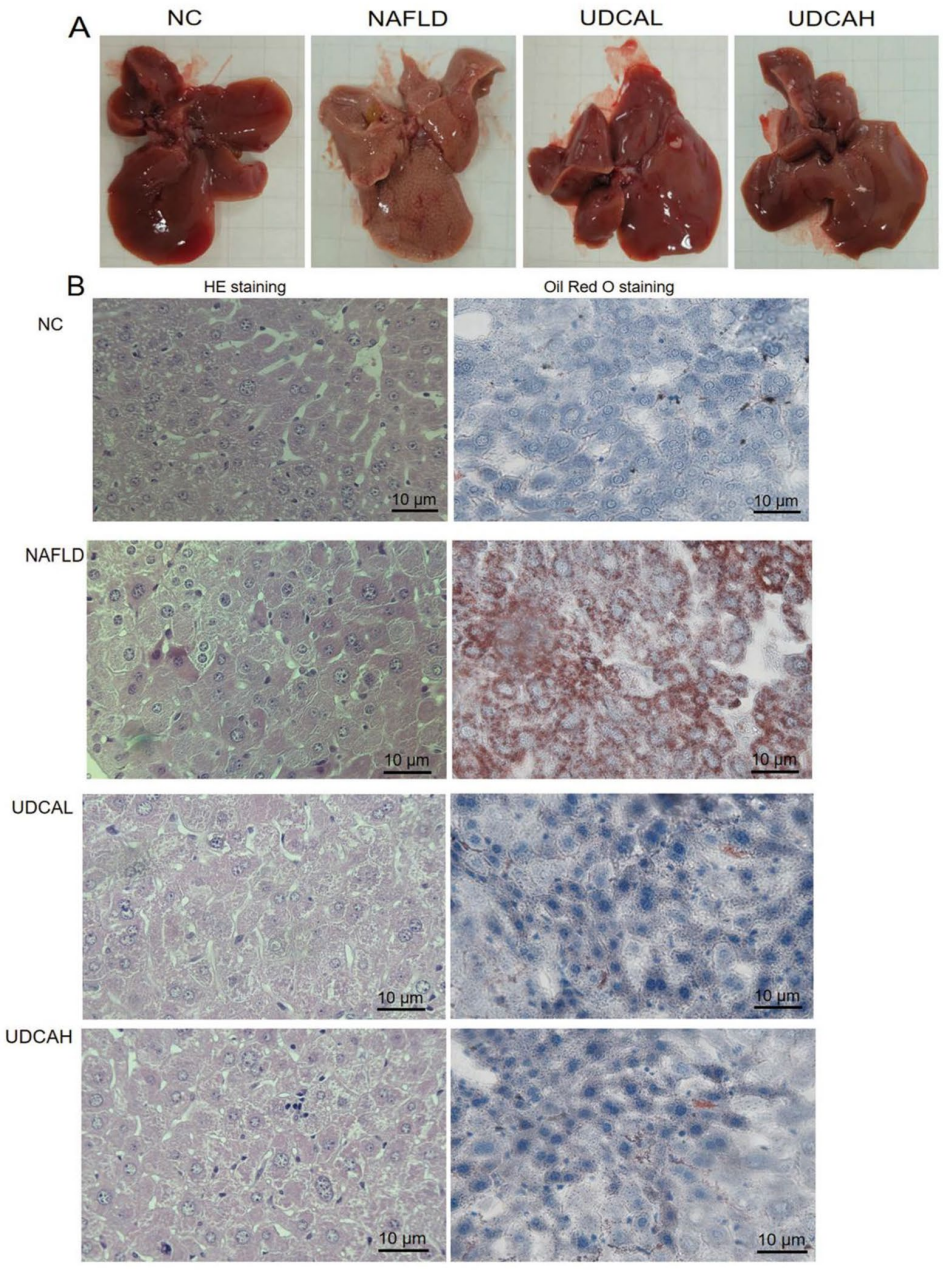
Representative images of hepatic pathology in mice. Gross morphological features of the liver **(A)** were assessed alongside histopathological examination using hematoxylin and eosin (HE) staining and lipid accumulation analysis *via* Oil Red O staining **(B)**.

Gross examination revealed that livers from normal control (NC) mice exhibited a dark reddish-brown coloration with soft, elastic texture and smooth surfaces devoid of greasiness, whereas NAFLD mice displayed markedly enlarged, pale yellow livers with thickened parenchyma and diffuse fatty sheen. High and low concentration UDCA (UDCAL and UDCAH groups) treatment substantially improved hepatic morphology, evidenced by partial restoration of liver color, elasticity, and reduced surface greasiness (Figure 2). HE staining demonstrated intact radial arrangement of hepatocyte cords in NC group, with well-defined central vein-portal triads architecture and uniformly distributed hepatocytes featuring large round nuclei and distinct nucleoli, devoid of vacuolar degeneration. In contrast, NAFLD livers exhibited widespread cytoplasmic vacuolation (lipid droplet dissolution artifacts), hepatocyte ballooning with peripherally displaced nuclei, disrupted cord architecture, and notable neutrophilic/lymphocytic infiltration. High and low concentration UDCA intervention mitigated these pathological changes, reducing lipid vacuolation, partially restoring hepatocyte cord organization, and decreasing inflammatory infiltrates (Figure 2). Oil Red O staining confirmed minimal microvesicular lipid droplets in NC hepatocytes versus NAFLD group’s abundant macrovesicular orange-red droplets compressing nuclei. High and low concentration UDCA treatment significantly attenuated lipid accumulation, predominantly maintaining microvesicular droplet distribution (Figure 2). These findings collectively demonstrate UDCA’s efficacy in counteracting diet-induced steatosis and preserving hepatic cytoarchitecture.

### Network pharmacology and molecular docking reveal the mechanism of UDCA in NAFLD treatment

**Figure 3:**
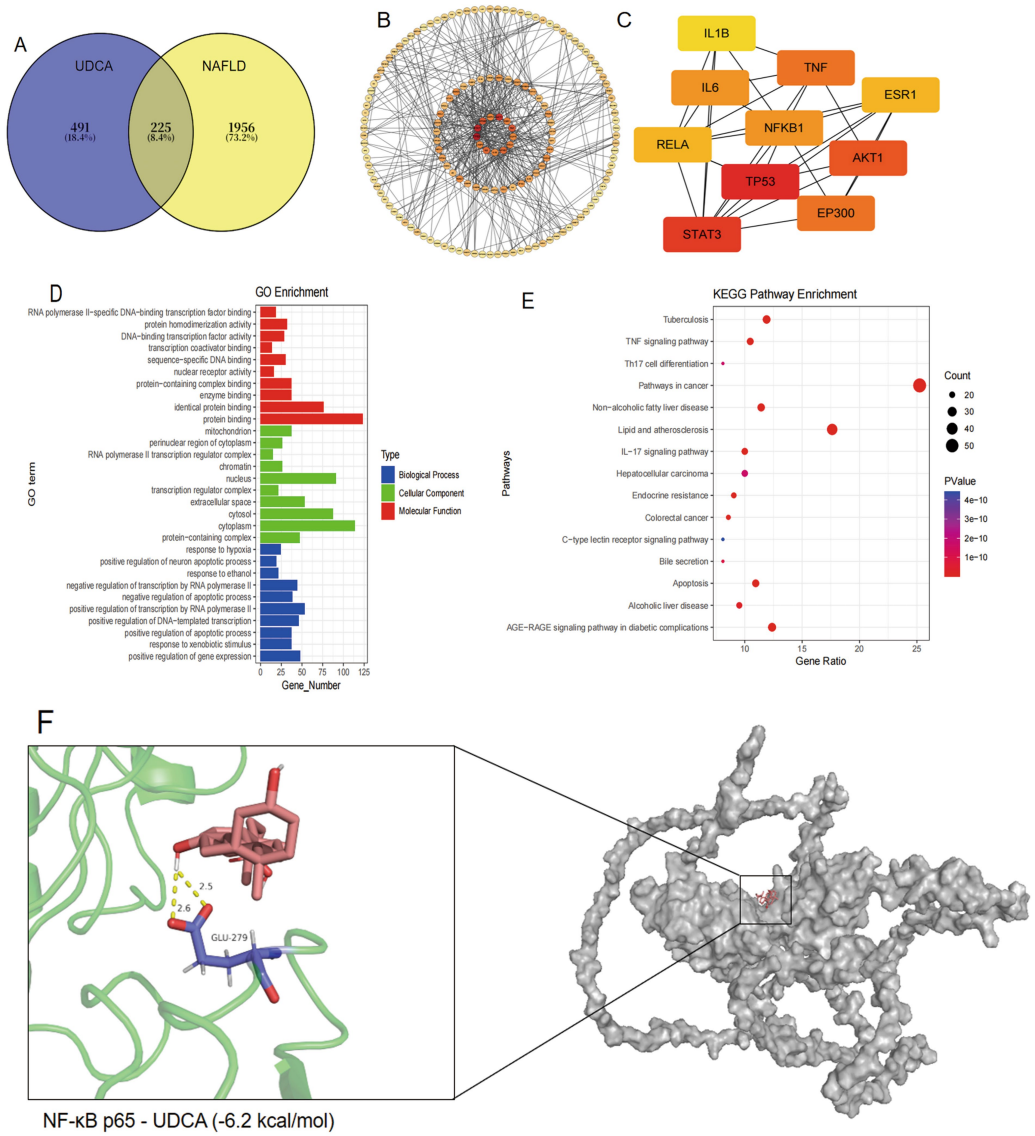
Network pharmacology and molecular docking analysis revealing the therapeutic mechanism of UDCA in NAFLD. **(A)** Venn diagram illustrating the overlapping targets between UDCA and NAFLD. **(B)** PPI network of the shared targets. **(C)** Core therapeutic targets with highest network centrality for UDCA treatment of NAFLD. **(D)** GO enrichment analysis showing significantly enriched biological processes, cellular components and molecular functions. **(E)** KEGG pathway enrichment analysis. **(F)** Molecular docking confirmation of binding interaction between UDCA and the NF-κB p65 protein target.

Comprehensive target identification was performed through multi-database mining, yielding 2,181 disease-related targets after merging results from TTD (23 targets), OMIM (200 targets), and GeneCards (2029 targets) with duplicate removal. Parallel screening of drug targets identified 716 UDCA-associated targets from PharmMapper (283 targets), SwissTarget Prediction (95 targets), and Comparative Toxicogenomics Database (367 targets). Integrated analysis revealed 225 overlapping therapeutic targets (Figure 3A) at the drug-disease interface, providing a systematic framework for mechanistic investigation of UDCA against NAFLD.

The 225 overlapping therapeutic targets were analyzed using the STRING database to construct a PPI network with high-confidence edges (interaction score ≥0.9). Visualization and topological analysis through Cytoscape software generated a network comprising 206 nodes and 527 edges (Figure 3B), where node color intensity reflected degree centrality values. Subsequent topological analysis identified key hub targets (TP53, STAT3, AKT1, TNF-*α*, EP300, NF-κB, and IL6) with the highest network connectivity (Figure 3C), suggesting their potential central roles in UDCA’s therapeutic mechanism against NAFLD.

Functional annotation of UDCA’s potential therapeutic targets was performed using the DAVID database, yielding 210 biological process terms, 210 cellular component terms, and 208 molecular function terms (Figure 3D). The most significantly enriched biological processes included positive regulation of gene expression, response to xenobiotic stimulus, and positive regulation of apoptotic process. Cellular components were primarily associated with protein-containing complexes, cytoplasm, and cytosol, while molecular functions predominantly involved protein binding, identical protein binding, and enzyme binding. KEGG pathway analysis identified 188 significantly enriched signaling pathways (*p* < 0.05), among which the most prominent were lipid and atherosclerosis, pathways in cancer, and the AGE-RAGE signaling pathway in diabetic complications (Figure 3E), suggesting UDCA’s potential multi-faceted mechanisms in NAFLD intervention. Molecular docking simulations demonstrate that ursodeoxycholic acid (UDCA) binds to the pocket of the NF-κB p65 protein with a binding affinity of –6.2 kcal/mol. This interaction is primarily stabilized by hydrogen bonds between UDCA and the GLU-279 residue (Figure 3F).

### UDCA alleviates hepatic steatosis and inflammation through PPARγ/Nrf2/NF-κB pathway

**Figure 4:**
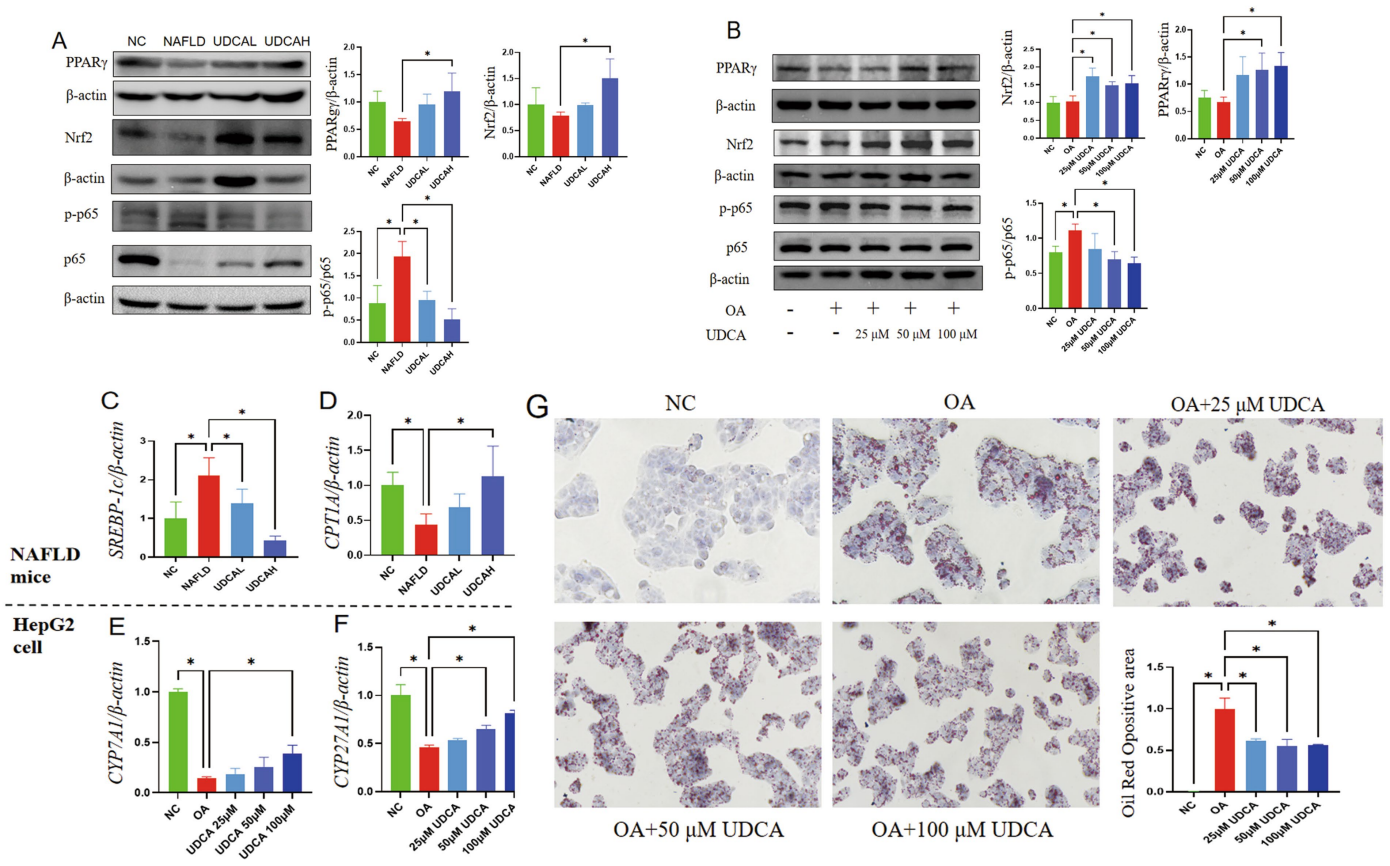
Validation of the Nrf2/PPARγ/NF-κB pathway *in vivo* and *in vitro* (*n* = 3). **(A)** Representative Western blot and quantitative analysis of hepatic PPARγ/β-actin, Nrf2/β-actin, and p-p65/p65 protein levels in mice. **(B,C)** Hepatic mRNA expression levels of SREBP-1c and CPT1A in experimental mice. **(D,E)** mRNA expression of CYP7A1 and CYP27A1 in HepG2 cells. **(F)** Representative Oil Red O staining images demonstrating intracellular lipid accumulation in HepG2 cells. ^*^p < 0.05 versus the respective control group. **(G)** Ursodeoxycholic acid (UDCA) attenuates oleic acid (OA)-induced lipid accumulation in cells. Representative images of Oil Red O staining and quantitative analysis of the Oil Red O positive area are shown. Cells were treated with OA to induce lipid accumulation and co-treated with indicated concentrations of UDCA (25, 50, and 100 μm). NC indicates the negative control group. Red staining indicates intracellular lipid droplets. The bar graph represents the quantification of the Oil Red O positive area. The results demonstrate that UDCA significantly reduced lipid accumulation compared to the OA group. (^*^p < 0.05).

Western blot analysis demonstrated that UDCA treatment significantly modulated the PPARγ/Nrf2/NF-κB pathway involved in hepatic lipid metabolism and inflammation (*in vivo* and *in vitro*). In NAFLD mice, UDCA administration markedly upregulated the lipid metabolism regulator PPARγ (*p* < 0.05), enhanced the expression of the antioxidant stress protein Nrf2 (*p* < 0.05), and reduced the phosphorylation level of NF-κB p65 (pp65) (*p* < 0.05) (Figure 4A), suggesting improved adipocyte differentiation and lipid homeostasis. Consistently, *in vitro* experiments in OA-induced HepG2 cells revealed that UDCA (50 μM and 100 μM) significantly increased Nrf2 and PPARγ expression while suppressing p65 phosphorylation (*p* < 0.05) (Figure 4B). These findings indicate that UDCA ameliorates hepatic steatosis and inflammation in NAFLD by enhancing lipid metabolism, mitigating oxidative stress, and inhibiting NF-κB-mediated inflammatory responses.

Quantitative PCR analysis revealed that UDCA treatment significantly downregulated hepatic SREBP-1 mRNA expression while upregulating CPT1A in NAFLD mice (*p* < 0.05) (Figures 4C,D), indicating improved lipid metabolism regulation. *In vitro* studies demonstrated that oleic acid (OA) suppressed both classical (CYP7A1) and alternative (CYP27A1) bile acid synthesis pathway enzymes in HepG2 cells (*p* < 0.05). Notably, high-dose UDCA (100 μM) effectively restored CYP7A1 and CYP27A1 expression levels (*p* < 0.05 vs. OA group) (Figures 4E,F), suggesting UDCA-mediated modulation of bile acid homeostasis in lipid-loaded hepatocytes.

Oil Red O staining revealed significant intracellular lipid accumulation in oleic acid (OA)-treated HepG2 cells compared to normal controls (*p* < 0.01) (Figure 4G). UDCA treatment demonstrated dose-dependent lipid-lowering effects, with 25 μM (low), 50 μM (medium), and 100 μM (high) concentrations all significantly reducing lipid droplet deposition (*p* < 0.05 vs. OA group). The most pronounced reduction was observed at the highest concentration (100 μM UDCA; *p* < 0.05), suggesting a potential therapeutic dose–response relationship.

### UDCA modulates serum bile acid profile and transport pathways

**Figure 5:**
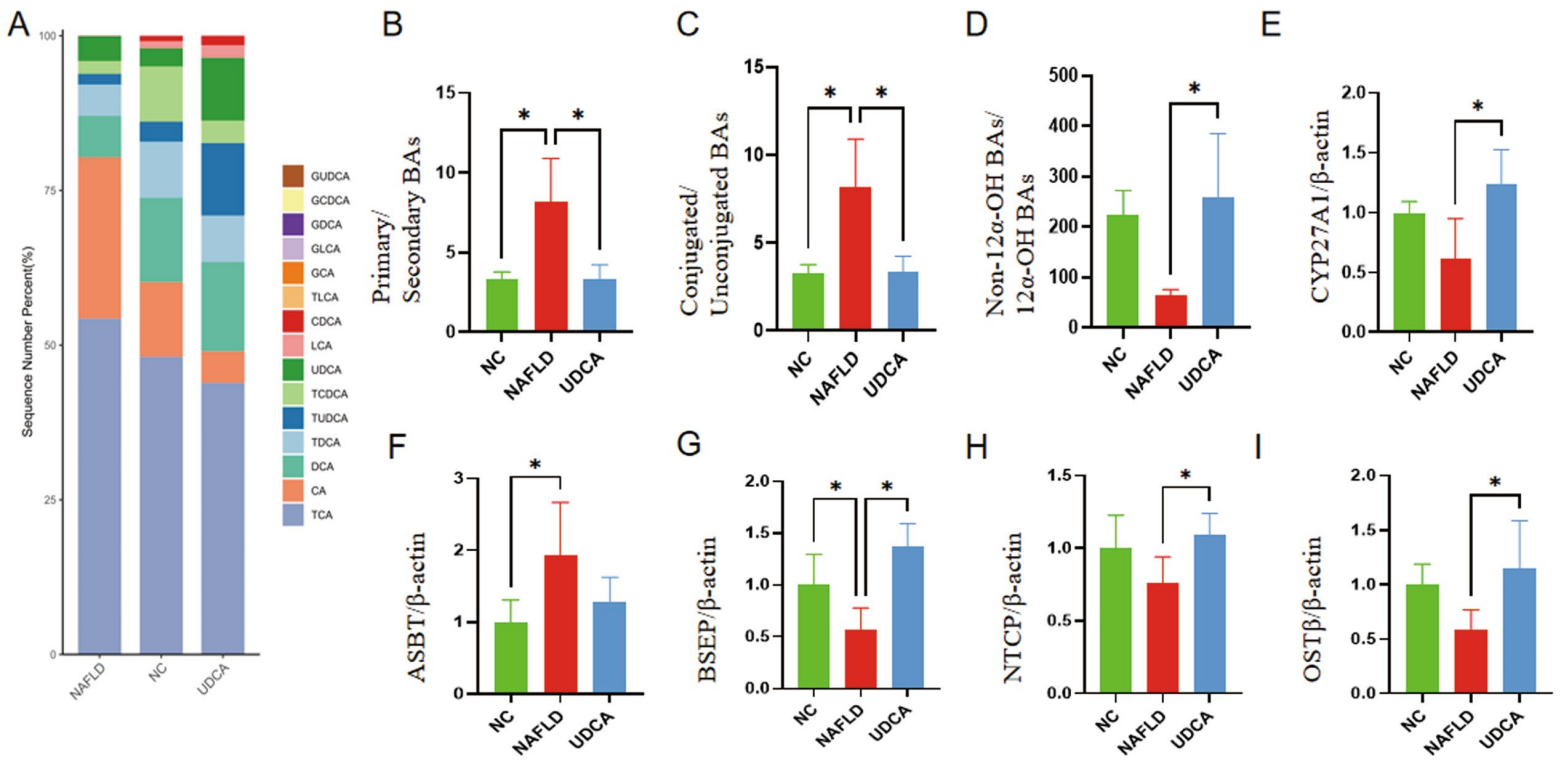
Comprehensive analysis of serum bile acid profiles and hepatic bile acid transporter expression in mice (*n* = 3). The figure presents: quantitative serum bile acid composition **(A)**, metabolic ratios of primary-to-secondary bile acids **(B)**, conjugated-to-unconjugated bile acids **(C)**, and non-12α-hydroxylated-to-12α-hydroxylated bile acids **(D)**, along with hepatic expression of CYP27A1 **(E)**. Transporter analysis includes ileal apical sodium-dependent bile acid transporter ASBT **(F)**, hepatic bile salt export pump BSEP **(G)**, sodium/taurocholate cotransporting polypeptide NTCP **(H)**, and ileal organic solute transporter β OSTβ, **(I)** mRNA expression. Statistically significant differences (*p* < 0.05) are indicated by asterisks (*).

This study found that a high dose of UDCA (30 mg/kg/day) exhibited superior therapeutic efficacy. The following research investigates the treatment mechanisms using this high-dose UDCA regimen. UPLC-MS/MS analysis revealed significant alterations in the serum bile acid profile following UDCA treatment: compared to NAFLD mice, the UDCA group exhibited marked reductions in taurocholic acid (TCA) and cholic acid (CA) (*p* < 0.05), alongside elevated levels of UDCA, chenodeoxycholic acid (CDCA), and tauroursodeoxycholic acid (TUDCA) (Figure 5A). Notably, NAFLD mice displayed significantly increased ratios of primary/secondary bile acids and conjugated/unconjugated bile acids relative to normal controls, both of which were attenuated by UDCA intervention (*p* < 0.05) (Figures 5B,C). Further metabolomic profiling demonstrated higher non-12*α*-OH bile acid levels in the UDCA-treated group versus NAFLD mice, suggesting potential activation of the alternative bile acid synthesis pathway (Figure 5D). Mechanistic validation via RT-qPCR showed upregulated hepatic expression of CYP27A1—a key enzyme in the alternative synthesis pathway—confirming UDCA-mediated modulation of bile acid metabolism (Figure 5E). These findings collectively indicate that UDCA not only reshapes the bile acid pool composition but also reprograms synthesis pathways through CYP27A1 induction, highlighting its therapeutic role in restoring bile acid homeostasis during NAFLD progression.

Quantitative RT-PCR analysis of hepatic bile acid transporters (Figures 3F–I) revealed significant dysregulation in NAFLD mice compared to NC group, with upregulated expression of the ileal apical sodium-dependent bile acid transporter (ASBT) (*p* < 0.05) and downregulated bile salt export pump (BSEP) (*p* < 0.05). Following UDCA treatment, while no significant reduction in ASBT was observed (*p* > 0.05), the expression of key hepatocyte transporters, including BSEP, sodium taurocholate co-transporting polypeptide (NTCP), and organic solute transporter *β* (OSTβ), was markedly increased (*p* < 0.05). These findings suggest that UDCA selectively enhances efflux transporters (BSEP, OSTβ) and uptake mechanisms (NTCP), potentially promoting bile acid clearance and ameliorating cholestatic features in NAFLD.

### UDCA modulates gut microbiota composition in NAFLD mice

**Figure 6:**
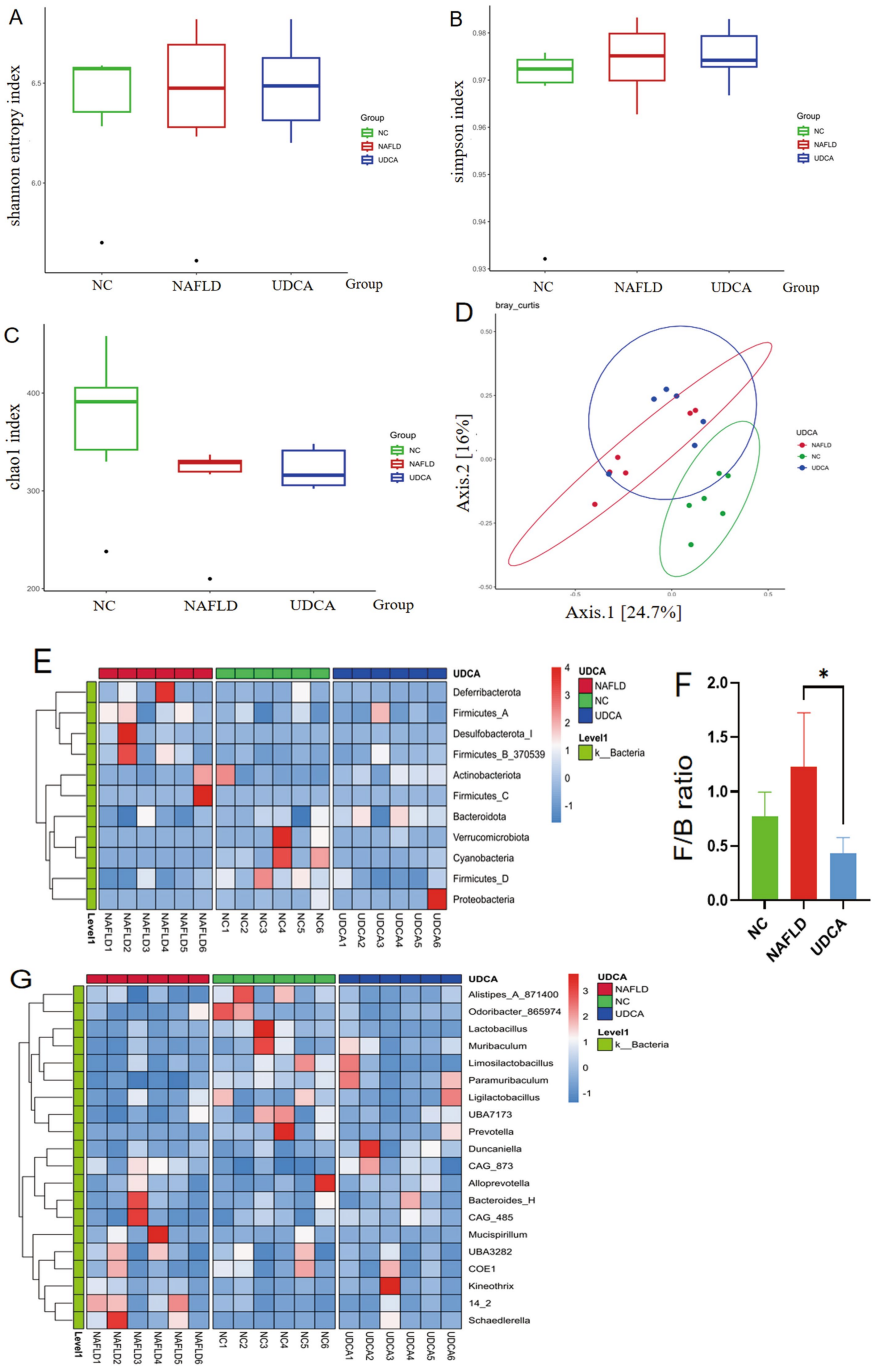
Results of 16S rRNA sequencing analysis of murine gut microbiota (*n* = 6). α-diversity assessments Shannon index **(A)**, Simpson index **(B)**, and Chao1 index **(C)** demonstrated no significant intergroup differences, whereas β-diversity analysis *via* principal coordinates analysis (PCoA) revealed clear separation between the NC group and both NAFLD and UDCA treatment groups **(D)**. Taxonomic profiling at phylum **(E)** and genus levels **(F)** Ursodeoxycholic acid (UDCA) reverses the elevated Firmicutes/Bacteroidetes (F/B) ratio in the gut microbiota. **(G)** showed altered microbial composition, with UDCA treatment significantly modulating the Firmicutes/Bacteroidetes (F/B) ratio compared to NAFLD controls. ^*^p < 0.05.

No significant alterations in gut microbial *α*-diversity (assessed *via* Shannon, Simpson, and Chao1 indices) were observed among the NC, NAFLD, and UDCA-treated groups, suggesting that neither NAFLD induction nor UDCA intervention affected overall microbial richness or evenness. However, principal coordinates analysis (PCoA) based on Bray–Curtis dissimilarity revealed distinct clustering patterns among the three groups, with significant separation between NC and NAFLD samples, confirming successful NAFLD model establishment. These results demonstrate that while α-diversity remained unchanged, UDCA treatment induced β-diversity shifts in colonic microbiota composition (Figures 6A–D).

**Figure 7:**
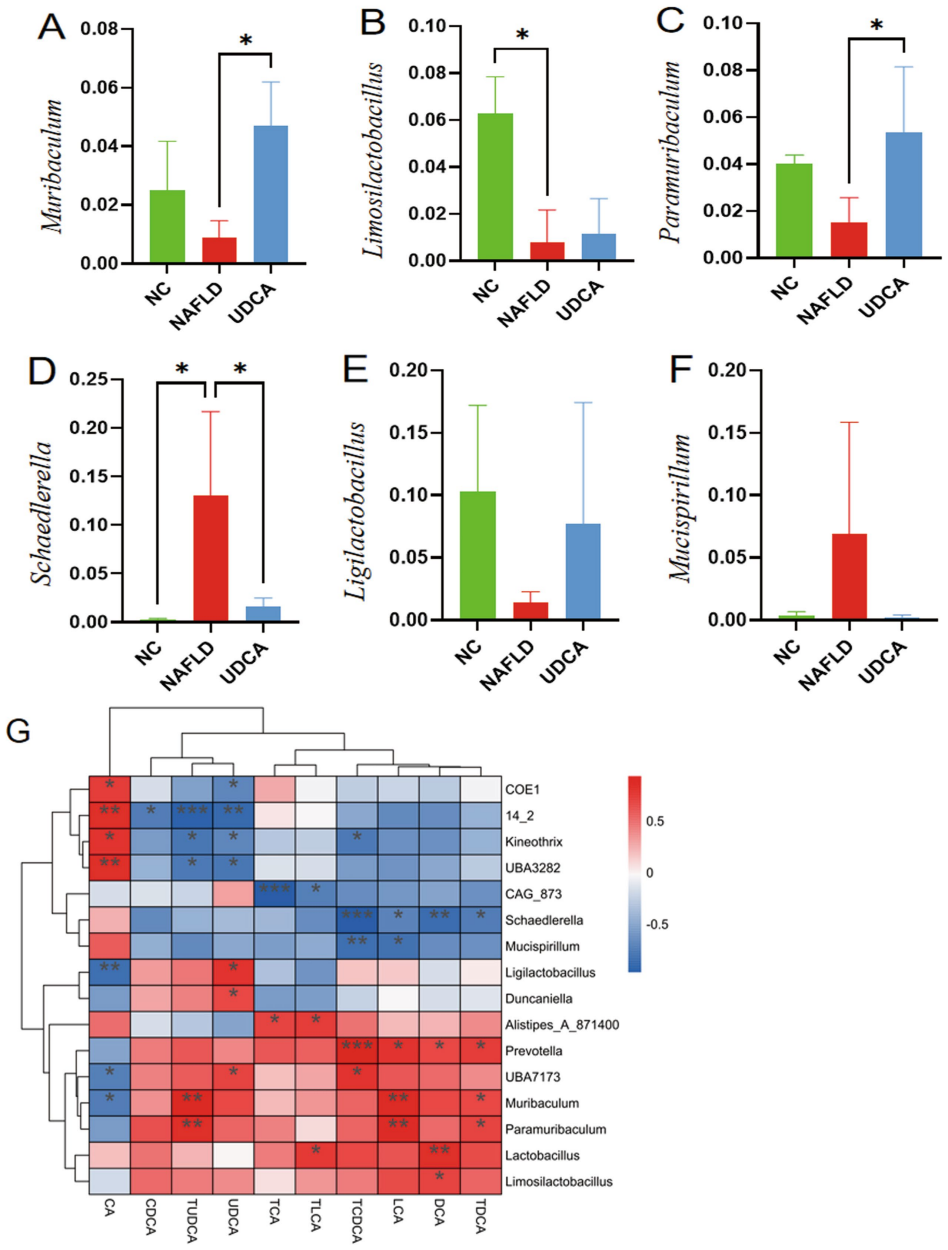
Alterations in genus-level gut microbiota composition and Spearman correlation analysis between serum bile acids and intestinal bacteria (*n* = 6). **(A–F)** Relative abundances of key genera: *Muribaculum* **(A)**, *Limosilactobacillus* **(B)**, *Paramuribaculum* **(C)**, *Schaedlerella* **(D)**, *Ligilactobacillus* **(E)**, and *Mucispirillum* **(F)**. **(G)** Spearman correlation heatmap illustrating the associations between serum bile acid profiles and gut microbial taxa. *p < 0.05; **p < 0.01; ***p < 0.001.

At the phylum level, NAFLD mice exhibited significantly increased abundance of Firmicutes, Deferribacterota, and Desulfobacterota compared to NC controls, while UDCA treatment reversed these changes and concurrently elevated Bacteroidota and Proteobacteria populations (Figure 6E). Notably, the Firmicutes/Bacteroidota (F/B) ratio in UDCA-treated animals was significantly lower than in NAFLD mice (*p* < 0.05) (Figure 6F), suggesting UDCA may exert therapeutic effects through Bacteroidota-promoting microbiota restructuring. Genus-level analysis revealed NAFLD-associated depletion of *Limosilactobacillus* and enrichment of *Schaedlerella* relative to NC mice. UDCA intervention significantly increased *Muribaculum* and *Paramuribaculum* abundance while reducing *Schaedlerella* colonization (*p* < 0.05) (Figures 6G, 7A–F), demonstrating compound-specific modulation of microbial subpopulations.

To elucidate potential pharmacological interactions between bile acids and gut microbiota, we performed systematic Spearman’s rank correlation analyses between specific bile acids and differentially abundant bacterial genera (Figure 7G). The analysis revealed significant positive correlations between TUDCA and *Paramuribaculum* (*r* = 0.62, *p* < 0.01), as well as between UDCA and *UBA7173* (*r* = 0.58, *p* < 0.05). Conversely, TCDCA showed significant negative correlations with *Schaedlerella* (*r* = −0.65, *p* < 0.01) and *Mucispirillum* (*r* = −0.53, *p* < 0.01). These findings demonstrate compound-specific modulatory relationships between secondary bile acids and gut microbiota populations, with particularly strong associations observed for TUDCA and TCDCA.

### UDCA treatment ameliorated metabolic disturbances in NAFLD mice

**Figure 8:**
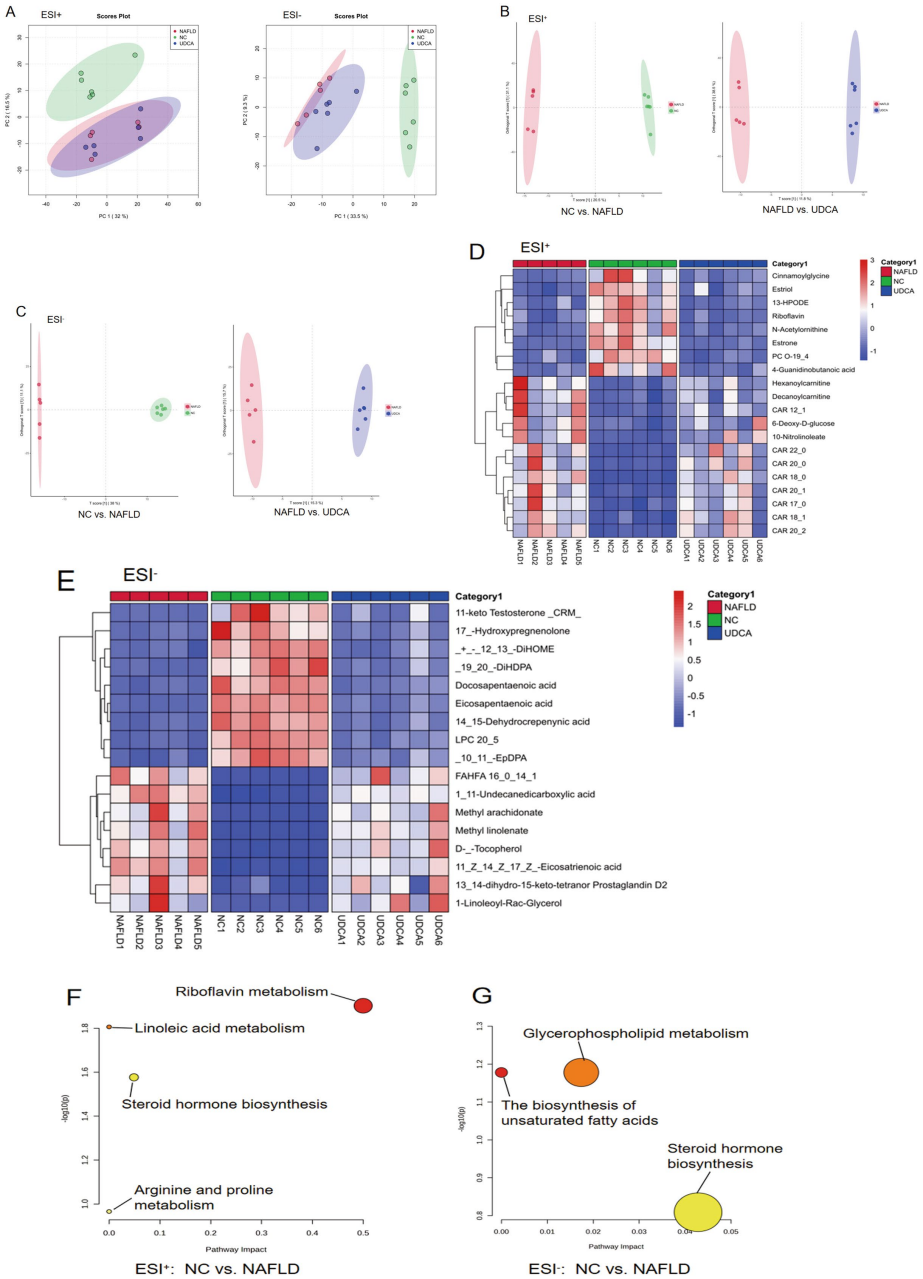
Metabolic profiling and pathway analysis of serum samples from mice (*n* = 6). Multivariate analysis of serum metabolites was performed using PCA **(A)** and OPLS-DA **(B,C)** in both ESI + and ESI- modes, demonstrating distinct metabolic clustering among experimental groups. Heatmaps **(D,E)** of significantly altered serum metabolites (VIP > 1, *p* < 0.05) in ESI + and ESI- modes further highlight the metabolic reprogramming induced by UDCA treatment. Pathway enrichment **(F,G)** analysis revealed the most significantly affected metabolic pathways (ESI + and ESI− modes), providing mechanistic insights into UDCA-mediated therapeutic effects.

Untargeted metabolomics analysis of serum samples from NC, NAFLD, and UDCA-treated groups revealed distinct metabolic profiles through PCA, with clear separation among groups in both ESI + and ESI- modes (Figure 8A). The pronounced segregation between NC and NAFLD groups demonstrated successful induction of metabolic dysfunction in the NAFLD model. Notably, UDCA-treated samples in ESI- mode exhibited clustering closer to NC controls relative to NAFLD mice, suggesting partial restoration of metabolic homeostasis. OPLS-DA further confirmed these findings, showing excellent model validity (NC vs. NAFLD: R2Y = 0.92, Q2 = 0.85; UDCA vs. NAFLD: R2Y = 0.88, Q2 = 0.79). The OPLS-DA score plots demonstrated marked separation between NC and NAFLD groups (Figures 8B,C), while UDCA-treated samples displayed significant directional shifts toward NC controls, indicating substantial amelioration of NAFLD-associated metabolic derangements.

Based on OPLS-DA analysis with selection criteria of VIP > 1 and *p* < 0.05 (Figures 8D,E; Supplementary Tables S2, S3), we identified a panel of statistically significant differential metabolites. The UDCA-treated group exhibited marked upregulation of anti-inflammatory and antioxidant metabolites, including eicosapentaenoic acid, coupled with significant downregulation of long-chain acylcarnitines (CAR 20:0). To elucidate the metabolic regulatory mechanisms of UDCA, we performed pathway enrichment analysis using MetaboAnalyst 5.0.^5^ The results demonstrated that UDCA intervention predominantly affected steroid hormone biosynthesis, caffeine metabolism, the pentose phosphate pathway, and pyrimidine metabolism (Figures 8A,B). These findings suggest that the therapeutic effects of UDCA in NAFLD are mediated through the modulation of these key metabolic pathways, particularly by ameliorating oxidative stress and improving lipid metabolic dysfunction. The coordinated regulation of pro-inflammatory mediators and oxidative-stress related metabolic networks provides mechanistic insight into UDCA’s pharmacological activity against NAFLD progression.

## Discussion

NAFLD, the most prevalent chronic liver disease worldwide, is characterized by a complex pathological interplay of metabolic dysfunction, cellular injury, inflammatory cascades, and progressive fibrosis, rendering single-target therapeutic strategies largely ineffective in halting disease progression (33, 34). Current clinical management remains limited to symptomatic relief, often challenged by insufficient hepatic targeting and long-term safety concerns (35, 36). While historically sourced from bear bile in traditional Chinese medicine, UDCA is now predominantly synthesized pharmaceutically, yet retains the holistic therapeutic advantages of its natural origin through multi-target regulation of metabolic and inflammatory pathways (24, 37). As a natural FXR modulator, UDCA not only ameliorates diet-induced dyslipidemia and hepatic steatosis but also normalizes serum ALT/AST levels by suppressing Kupffer cell activation and exhibits antifibrotic potential (38–40). Our 8-week intervention study (30 mg/kg/day, oral gavage) further revealed UDCA’s dual metabolic modulation—concurrently activating PPARγ to enhance lipid oxidation while inhibiting SREBP-1c to reduce lipogenesis—thereby mechanistically elucidating its traditional “liver-soothing and bile-promoting” effects (41–43). Remarkably, UDCA rebalanced the gut microbiota in NAFLD mice by correcting the Firmicutes/Bacteroidetes (F/B) ratio, suggesting a microbiota-liver axis-mediated protective mechanism. With superior biocompatibility due to its natural origin and inherent liver-targeting properties, UDCA offers distinct advantages over synthetic drugs. These findings provide a strong rationale for developing integrated therapies combining UDCA with modern pharmacological approaches.

As central regulators of hepatic lipid metabolism, bile acids exhibit disrupted homeostasis closely associated with NAFLD pathogenesis (44, 45). This study elucidates the hepatoprotective mechanisms of UDCA through remodeling of the bile acid metabolic profile and dynamic regulation of bile acid transport. Specifically, UDCA treatment significantly increased the proportion of non-12α-hydroxy bile acids (non-12α-OH BAs). This metabolic shift from classical to alternative synthesis pathways—previously shown to ameliorate metabolic disorders by reducing hepatic triglyceride content and lipid accumulation—was strongly corroborated by our experimental results (18, 46, 47). Mechanistic investigations revealed that NAFLD-induced bile acid dysregulation manifests as hepatic cholestasis resulting from both impaired biliary excretion (*via* decreased bile salt output) and enhanced ileal reabsorption, collectively exacerbating hepatocellular injury (48, 49). UDCA intervention dynamically modulated key transporters through three coordinated actions: (1) upregulation of the canalicular bile acid exporter BSEP to enhance hepatocellular excretion (50, 51), (2) downregulation of the ileal bile acid uptake transporter OST*β* to limit intestinal reabsorption (52, 53), and (3) increased expression of the portal vein uptake transporter NTCP to reduce systemic bile acid levels (28, 54). This multi-target synergy effectively alleviated hepatic bile acid overload and mitigated systemic bile acid toxicity, providing a robust pharmacological rationale for UDCA’s therapeutic application in NAFLD management.

The gut microbiota plays a pivotal role in maintaining host metabolic homeostasis, with its dysbiosis being firmly associated with various metabolic disorders including NAFLD (55). Our study demonstrates that UDCA administration significantly ameliorates gut microbial dysregulation in NAFLD mice through three key modulations: (1) marked enrichment of *Muribaculum* spp., which ferment dietary fiber and endogenous mucin glycans to produce short-chain fatty acids while establishing cross-feeding networks with beneficial genera (*Bifidobacterium*, *Lactobacillus*), where its abundance increase correlates with metabolic improvement in conditions like obesity and type 2 diabetes (56, 57); (2) promotion of probiotic proliferation (*Limosilactobacillus*, *Ligilactobacillus*) to reinforce intestinal microenvironment homeostasis (58, 59); and (3) significant reduction of the opportunistic pathogen *CAG-485* (a *Prevotella* species) known to exacerbate intestinal inflammation and systemic immune imbalance *via* mucus layer degradation and IL-18 suppression (60, 61). Notably, correlation analysis revealed strong positive associations between these beneficial taxa and secondary bile acids (TUDCA, UDCA), further validating UDCA’s therapeutic mechanism through the microbiota-bile acid metabolic axis. Collectively, beyond direct hepatic lipid metabolism regulation, UDCA mitigates NAFLD progression through multidimensional mechanisms including gut microbiota restructuring, intestinal barrier reinforcement, and systemic proinflammatory factor reduction.

Serum metabolomics confirmed our observations by demonstrating that UDCA treatment partially restored the metabolic profile of NAFLD mice toward normal conditions. Comparative analysis revealed significant alterations in circulating metabolites, with UDCA-treated mice exhibiting greater similarity to healthy controls. Notably, UDCA intervention significantly reduced serum levels of long-chain acylcarnitines (LCACs, e.g., CAR18:0), whose accumulation reflects impaired mitochondrial *β*-oxidation and actively contributes to mitochondrial dysfunction through a vicious cycle (62, 63). This reduction was mechanistically linked to enhanced Nrf2-mediated antioxidant defenses and upregulated CPT1A expression, thereby improving incomplete fatty acid oxidation associated with obesity and insulin resistance (64, 65). Concurrently, UDCA treatment elevated circulating levels of omega-3 polyunsaturated fatty acids (EPA and DPA), which function as endogenous PPARγ agonists (66, 67). Through PPARγ activation, these metabolites orchestrate a comprehensive metabolic improvement by: (1) reducing hepatic free fatty acid influx (68), (2) enhancing mitochondrial β-oxidation capacity (69), (3) attenuating oxidative stress and inflammation (70), and (4) promoting gut barrier integrity (71). These findings establish UDCA as a multifaceted regulator of mitochondrial and lipid metabolism in NAFLD pathophysiology.

To further validate the molecular mechanisms observed *in vivo*, we employed an OA-induced HepG2 cellular model of NAFLD. UDCA treatment significantly attenuated lipid accumulation, demonstrating its direct hepatoprotective effects. Mechanistically, UDCA activated PPARγ, which suppressed NF-κB-mediated inflammatory responses (72), thereby reducing OA-induced pro-inflammatory signaling (73). Additionally, Nrf2 activation by UDCA enhanced cellular antioxidant capacity, counteracting oxidative stress—a key driver of NAFLD progression (7). These findings indicate that UDCA orchestrates a coordinated defense against lipotoxicity through dual modulation of Nrf2 (antioxidant) and PPARγ/NF-κB (anti-inflammatory) pathways, reinforcing its therapeutic potential in metabolic liver diseases.

In summary, our study demonstrates that UDCA effectively ameliorates NAFLD through a multi-tiered mechanism involving (1) modulation of serum bile acid profiles, (2) restoration of gut microbiota homeostasis, and (3) systemic metabolic improvements, ultimately activating Nrf2-mediated antioxidant responses and PPARγ-dependent anti-inflammatory signaling while suppressing NF-κB phosphorylation. However, this work has limitations that warrant consideration: First, while serum bile acid profiling revealed significant alterations, complementary analysis of hepatic and fecal bile acids would provide deeper insights into UDCA’s impact on bile acid synthesis and enterohepatic circulation. Second, although microbiota restructuring correlated with metabolic benefits, causality remains unproven; future fecal microbiota transplantation (FMT) studies are needed to establish direct microbial contributions. Third, despite a modest sample size (10 per group), the consistent NAFLD phenotype across all HFD-fed mice (confirmed by histopathology) supports the reliability of our findings. These limitations highlight key directions for further investigation while reinforcing UDCA’s potential as a multi-target therapy for NAFLD.

## Conclusion

Our integrative multi-omics investigation establishes UDCA as a pleiotropic modulator of the gut-liver axis in NAFLD pathogenesis, demonstrating three synergistic mechanisms of action: (1) restoration of bile acid homeostasis through selective enrichment of hepatoprotective secondary bile acids (particularly TUDCA and unconjugated UDCA); (2) correction of HFD-induced microbial dysbiosis via significant reductions in the Firmicutes/Bacteroidetes ratio and expansion of SCFA-producing *Muribaculaceae* taxa; (3) direct targeting of NAFLD’s core pathological network evidenced by PPI analysis revealing UDCA’s modulation of central hub targets (TP53, STAT3, AKT1, TNF-*α*, NF-κB) and enrichment in lipid metabolism (PPARγ), inflammation (AGE-RAGE), and oxidative stress pathways; and (4) coordinated metabolic reprogramming through PPARγ-mediated enhancement of fatty acid oxidation coupled with Nrf2-dependent antioxidant responses, while simultaneously suppressing NF-κB-mediated inflammatory cascades. These findings provide compelling experimental evidence that substantiates the traditional application of bear bile derivatives in hepatobiliary disorders, while mechanistically elucidating UDCA’s unique capacity to concurrently address microbial dysregulation, metabolic dysfunction, and chronic inflammation—the pathogenic triad driving NAFLD progression. From a translational perspective, these results position UDCA as a promising candidate for adjunctive NAFLD therapy.

## Data Availability

The original contributions presented in the study are publicly available. This data can be found here: https://ngdc.cncb.ac.cn/gsa/browse/CRA036131.

## Footnotes

1
http://www.hmdb.ca/

2
https://metlin.scripps.edu/

3
https://www.kegg.jp/kegg/mapper.html

4
https://bioincloud.tech/

5
https://www.metaboanalyst.ca/

## References

[1] Lekakis V Papatheodoridis GV. Natural history of metabolic dysfunction-associated steatotic liver disease. Eur J Intern Med. (2024) 122:3–10. doi: 10.1016/j.ejim.2023.11.005, 37940495

[2] Sumida Y Yoneda M. Current and future pharmacological therapies for NAFLD/NASH. J Gastroenterol. (2018) 53:362–76. doi: 10.1007/s00535-017-1415-1, 29247356 PMC5847174

[3] Nassir F. NAFLD: mechanisms, treatments, and biomarkers. Biomolecules. (2022) 12:824. doi: 10.3390/biom12060824, 35740949 PMC9221336

[4] Pietrocola F Bravo-San Pedro JM. Targeting autophagy to counteract obesity-associated oxidative stress. Antioxidants. (2021) 10:102. doi: 10.3390/antiox10010102, 33445755 PMC7828170

[5] Chen Z Tian R She Z Cai J Li H. Role of oxidative stress in the pathogenesis of nonalcoholic fatty liver disease. Free Radic Biol Med. (2020) 152:116–41. doi: 10.1016/j.freeradbiomed.2020.02.025, 32156524

[6] Xu X Luo D Xuan Q Lu P Yu C Guan Q. Atlas of metabolism reveals palmitic acid results in mitochondrial dysfunction and cell apoptosis by inhibiting fatty acid β-oxidation in Sertoli cells. Front Endocrinol. (2022) 13:1021263. doi: 10.3389/fendo.2022.1021263, 36237186 PMC9552013

[7] Arroyave-Ospina JC Wu Z Geng Y Moshage H. Role of oxidative stress in the pathogenesis of non-alcoholic fatty liver disease: implications for prevention and therapy. Antioxidants. (2021) 10:174. doi: 10.3390/antiox10020174, 33530432 PMC7911109

[8] Guo X Yin X Liu Z Wang J. Non-alcoholic fatty liver disease (NAFLD) pathogenesis and natural products for prevention and treatment. Int J Mol Sci. (2022) 23:15489. doi: 10.3390/ijms232415489, 36555127 PMC9779435

[9] Feng X Yu W Li X Zhou F Zhang W Shen Q . Apigenin, a modulator of PPARγ, attenuates HFD-induced NAFLD by regulating hepatocyte lipid metabolism and oxidative stress via Nrf2 activation. Biochem Pharmacol. (2017) 136:136–49. doi: 10.1016/j.bcp.2017.04.014, 28414138

[10] Luo J Wang J Zhang J Sang A Ye X Cheng Z . Nrf2 deficiency exacerbated CLP-induced pulmonary injury and inflammation through autophagy- and NF-κB/PPARγ-mediated macrophage polarization. Cells. (2022) 11:3927. doi: 10.3390/cells11233927, 36497185 PMC9735993

[11] Grylls A Seidler K Neil J. Link between microbiota and hypertension: focus on LPS/TLR4 pathway in endothelial dysfunction and vascular inflammation, and therapeutic implication of probiotics. Biomed Pharmacother. (2021) 137:111334. doi: 10.1016/j.biopha.2021.111334 33556874

[12] Li W Huang D Luo Z Zhou T Jin Z. Yinchenhao decoction mitigates cholestatic liver injury in mice via gut microbiota regulation and activation of FXR-FGF15 pathway. Pharmaceuticals. (2025) 18:932. doi: 10.3390/ph18070932, 40732223 PMC12300480

[13] Schneider KM Candels LS Hov JR Myllys M Hassan R Schneider CV . Gut microbiota depletion exacerbates cholestatic liver injury via loss of FXR signalling. Nat Metab. (2021) 3:1228–41. doi: 10.1038/s42255-021-00452-1, 34552267

[14] Cao L Wu Y Liu K Qi N Zhang J Tie S. *Cornus officinalis* vinegar alters the gut microbiota, regulating lipid droplet changes in nonalcoholic fatty liver disease model mice. Food Med Homol. (2024) 1:9420002. doi: 10.26599/FMH.2024.9420002

[15] An C Chon H Ku W Eom S Seok M Kim S . Bile acids: major regulator of the gut microbiome. Microorganisms. (2022) 10:1792. doi: 10.3390/microorganisms10091792, 36144395 PMC9502002

[16] Lin Z Ma X. Dietary nutrients mediate crosstalk between bile acids and gut microbes in animal host metabolism. Crit Rev Food Sci Nutr. (2023) 63:9315–29. doi: 10.1080/10408398.2022.2067118, 35507502

[17] Su X Gao Y Yang R. Gut microbiota derived bile acid metabolites maintain the homeostasis of gut and systemic immunity. Front Immunol. (2023) 14:1127743. doi: 10.3389/fimmu.2023.1127743, 37256134 PMC10225537

[18] Collins SL Stine JG Bisanz JE Okafor CD Patterson AD. Bile acids and the gut microbiota: metabolic interactions and impacts on disease. Nat Rev Microbiol. (2023) 21:236–47. doi: 10.1038/s41579-022-00805-x, 36253479 PMC12536349

[19] Durník R Šindlerová L Babica P Jurček O. Bile acids transporters of enterohepatic circulation for targeted drug delivery. Molecules. (2022) 27:2961. doi: 10.3390/molecules27092961, 35566302 PMC9103499

[20] Jiang J Zhang H Hussain M Abdullah Feng F Guan R . Novel approaches in glucose and lipid metabolism disorder therapy: targeting the gut microbiota-bile acid axis. Biology. (2025) 14:802. doi: 10.3390/biology14070802 40723361 PMC12292970

[21] Jia W Li Y Cheung K Zheng X. Bile acid signaling in the regulation of whole body metabolic and immunological homeostasis. Sci China Life Sci. (2024) 67:865–78. doi: 10.1007/s11427-023-2353-0, 37515688

[22] Yang Z Danzeng A Liu Q Zeng C Xu L Mo J . The role of nuclear receptors in the pathogenesis and treatment of non-alcoholic fatty liver disease. Int J Biol Sci. (2024) 20:113–26. doi: 10.7150/ijbs.87305, 38164174 PMC10750283

[23] Pi Y Wu Y Zhang X Lu D Han D Zhao J . Gut microbiota-derived ursodeoxycholic acid alleviates low birth weight-induced colonic inflammation by enhancing M2 macrophage polarization. Microbiome. (2023) 11:19. doi: 10.1186/s40168-022-01458-x, 36721210 PMC9887892

[24] Song P Zhang X Feng W Xu W Wu C Xie S . Biological synthesis of ursodeoxycholic acid. Front Microbiol. (2023) 14:1140662. doi: 10.3389/fmicb.2023.1140662, 36910199 PMC9998936

[25] Zhang F Ju J Diao H Song J Bian Y Yang B. Innovative pharmacotherapy for hepatic metabolic and chronic inflammatory diseases in China. Br J Pharmacol. (2024) 182:4741–4760. doi: 10.1111/bph.16342, 38514420

[26] Li H Wang M Chen P Zhu M Chen L. A high-dose of ursodeoxycholic acid treatment alleviates liver inflammation by remodeling gut microbiota and bile acid profile in a mouse model of non-alcoholic steatohepatitis. Biomed Pharmacother. (2024) 174:116617. doi: 10.1016/j.biopha.2024.116617 38643542

[27] Qi Y Ma Y Duan G. Pharmacological mechanisms of bile acids targeting the farnesoid X receptor. Int J Mol Sci. (2024) 25:13656. doi: 10.3390/ijms252413656, 39769418 PMC11727972

[28] Yu H Nie R Shen C. The role of bile acids in regulating glucose and lipid metabolism. Endocr J. (2023) 70:359–74. doi: 10.1507/endocrj.EJ22-0544, 36928060

[29] Zhang Y Jiang R Zheng X Lei S Huang F Xie G . Ursodeoxycholic acid accelerates bile acid enterohepatic circulation. Br J Pharmacol. (2019) 176:2848–63. doi: 10.1111/bph.14705, 31077342 PMC6637225

[30] Hegazi OE Alalalmeh SO Shahwan M Jairoun AA Alourfi MM Bokhari GA . Exploring promising therapies for non-alcoholic fatty liver disease: a clinicalTrials.Gov analysis. Diabetes Metab Syndr Obes. (2024) 17:545–61. doi: 10.2147/DMSO.S448476, 38327733 PMC10847589

[31] Zhang X Coker OO Chu ES Fu K Lau H Wang YX . Dietary cholesterol drives fatty liver-associated liver cancer by modulating gut microbiota and metabolites. Gut. (2021) 70:761–74. doi: 10.1136/gutjnl-2019-319664, 32694178 PMC7948195

[32] Ren C-X Gao M-Y Li N Tang C Chu G-H Yusuf A. Identification and mechanism elucidation of medicative diet for food therapy XQCSY in NAFLD prevention: an integrative in silico study. Food Med. (2024) 1:9420015. doi: 10.26599/FMH.2024.9420015

[33] Trauner M Fuchs CD. Novel therapeutic targets for cholestatic and fatty liver disease. Gut. (2022) 71:194–209. doi: 10.1136/gutjnl-2021-324305, 34615727 PMC8666813

[34] Zhu S Wu Z Wang W Wei L Zhou H. A revisit of drugs and potential therapeutic targets against non-alcoholic fatty liver disease: learning from clinical trials. J Endocrinol Investig. (2024) 47:761–76. doi: 10.1007/s40618-023-02216-y 37839037

[35] Chen J Chua D Lim CO Ho WX Tan NS. Lessons on drug development: a literature review of challenges faced in nonalcoholic fatty liver disease (NAFLD) clinical trials. Int J Mol Sci. (2022) 24:158. doi: 10.3390/ijms24010158, 36613602 PMC9820446

[36] Mallet M Silaghi CA Sultanik P Conti F Rudler M Ratziu V . Current challenges and future perspectives in treating patients with NAFLD-related cirrhosis. Hepatology. (2024) 80:1270–90. doi: 10.1097/HEP.0000000000000456 37183906

[37] Li X Hu Y He B Li L Tian Y Xiao Y . Design, synthesis and evaluation of ursodeoxycholic acid-cinnamic acid hybrids as potential anti-inflammatory agents by inhibiting Akt/NF-κB and MAPK signaling pathways. Eur J Med Chem. (2023) 260:115785. doi: 10.1016/j.ejmech.2023.115785, 37678142

[38] Cavusoglu Nalbantoglu I Sevgi S Kerimoglu G Kadıoglu Duman M Kalyoncu NI. Ursodeoxycholic acid ameliorates erectile dysfunction and corporal fibrosis in diabetic rats by inhibiting the TGF-β1/Smad2 pathway. Int J Impot Res. (2024) 36:886–95. doi: 10.1038/s41443-024-00868-9, 38454160

[39] She J Gu T Pang X Liu Y Tang L Zhou X. Natural products targeting liver X receptors or farnesoid X receptor. Front Pharmacol. (2021) 12:772435. doi: 10.3389/fphar.2021.772435 35069197 PMC8766425

[40] Shimoyama S Kawata K Ohta K Chida T Suzuki T Tsuneyama K . Ursodeoxycholic acid impairs liver-infiltrating T-cell chemotaxis through IFN-γ and CX3CL1 production in primary biliary cholangitis. Eur J Immunol. (2021) 51:1519–30. doi: 10.1002/eji.202048589, 33710617

[41] Fontaine C Staels B. The orphan nuclear receptor rev-erbalpha: a transcriptional link between circadian rhythmicity and cardiometabolic disease. Curr Opin Lipidol. (2007) 18:141–6. doi: 10.1097/MOL.0b013e3280464ef6 17353661

[42] Lan T Geng XJ Zhang SJ Zeng XX Ying JJ Xu Y . Si-Ni-San inhibits hepatic Fasn expression and lipid accumulation in MAFLD mice through AMPK/p300/SREBP-1c axis. Phytomedicine. (2024) 123:155209. doi: 10.1016/j.phymed.2023.155209, 37984123

[43] Peng D Chen Y Sun Y Zhang Z Cui N Zhang W . Saikosaponin a and its epimers alleviate LPS-induced acute lung injury in mice. Molecules. (2023) 28:967. doi: 10.3390/molecules28030967, 36770631 PMC9919285

[44] Arab JP Karpen SJ Dawson PA Arrese M Trauner M. Bile acids and nonalcoholic fatty liver disease: molecular insights and therapeutic perspectives. Hepatology. (2017) 65:350–62. doi: 10.1002/hep.28709, 27358174 PMC5191969

[45] Grüner N Mattner J. Bile acids and microbiota: multifaceted and versatile regulators of the liver-gut axis. Int J Mol Sci. (2021) 22:1397. doi: 10.3390/ijms22031397, 33573273 PMC7866539

[46] Jia W Wei M Rajani C Zheng X. Targeting the alternative bile acid synthetic pathway for metabolic diseases. Protein Cell. (2021) 12:411–25. doi: 10.1007/s13238-020-00804-9, 33252713 PMC8106556

[47] Kuang J Wang J Li Y Li M Zhao M Ge K . Hyodeoxycholic acid alleviates non-alcoholic fatty liver disease through modulating the gut-liver axis. Cell Metab. (2023) 35:1752–1766.e8. doi: 10.1016/j.cmet.2023.07.011, 37591244

[48] Tian JY Xiao M Zhao WW Wu X Yang J Chen XQ. Effect of *Ilex hainanensis* Merr. On HFD-induced nonalcoholic fatty liver disease and rebalance of gut microbiota and bile acids metabolism in mice. Fitoterapia. (2024) 178:106186. doi: 10.1016/j.fitote.2024.106186 39142527

[49] Wei M Tu W Huang G. Regulating bile acids signaling for NAFLD: molecular insights and novel therapeutic interventions. Front Microbiol. (2024) 15:1341938. doi: 10.3389/fmicb.2024.1341938, 38887706 PMC11180741

[50] Kjærgaard K Frisch K Sørensen M Munk OL Hofmann AF Horsager J . Obeticholic acid improves hepatic bile acid excretion in patients with primary biliary cholangitis. J Hepatol. (2021) 74:58–65. doi: 10.1016/j.jhep.2020.07.028 32717289

[51] Wang X Xiong W Wang X Qin L Zhong M Liu Y . Ursolic acid attenuates cholestasis through NRF2-mediated regulation of UGT2B7 and BSEP/MRP2. Naunyn Schmiedeberg's Arch Pharmacol. (2024) 397:2257–67. doi: 10.1007/s00210-023-02733-w 37812240

[52] Calzadilla N Comiskey SM Dudeja PK Saksena S Gill RK Alrefai WA. Bile acids as inflammatory mediators and modulators of intestinal permeability. Front Immunol. (2022) 13:1021924. doi: 10.3389/fimmu.2022.1021924, 36569849 PMC9768584

[53] Wang Y Xu H Zhou X Chen W Zhou H. Dysregulated bile acid homeostasis: unveiling its role in metabolic diseases. Med Rev. (2024) 4:262–83. doi: 10.1515/mr-2024-0020, 39135605 PMC11317083

[54] Xue R Su L Lai S Wang Y Zhao D Fan J . Bile acid receptors and the gut-liver axis in nonalcoholic fatty liver disease. Cells. (2021) 10:2806. doi: 10.3390/cells10112806, 34831031 PMC8616422

[55] Fan Y Pedersen O. Gut microbiota in human metabolic health and disease. Nat Rev Microbiol. (2021) 19:55–71. doi: 10.1038/s41579-020-0433-9 32887946

[56] Wang N Dilixiati Y Xiao L Yang H Zhang Z. Different short-chain fatty acids unequally modulate intestinal homeostasis and reverse obesity-related symptoms in lead-exposed high-fat diet mice. J Agric Food Chem. (2024) 72:18971–85. doi: 10.1021/acs.jafc.4c04193, 39146036

[57] Wang Z Kang S Wu Z Liu X Zhang X Wu Y . *Muribaculum intestinale* restricts *Salmonella Typhimurium* colonization by converting succinate to propionate. ISME J. (2025) 19:wraf069. doi: 10.1093/ismejo/wraf069, 40249311 PMC12064562

[58] Yang J Shang P Liu Z Wang J Zhang B Zhang H. *Ligilactobacillus salivarius* regulating translocation of core bacteria to enrich mouse intrinsic microbiota of heart and liver in defense of heat stress. Front Immunol. (2025) 16:1540548. doi: 10.3389/fimmu.2025.1540548, 40276518 PMC12018310

[59] Zhou X Xu Q Zhang X Wang H Bai Y Wu Y . Mucin alleviates colonic barrier dysfunction by promoting spermine accumulation through enhanced arginine metabolism in Limosilactobacillus mucosae. mSystems. (2024) 9:e0024624. doi: 10.1128/msystems.00246-24 38564708 PMC11097634

[60] Beteri B Barone M Turroni S Brigidi P Tzortzis G Vulevic J . Impact of combined prebiotic galacto-oligosaccharides and *Bifidobacterium breve*-derived postbiotic on gut microbiota and HbA1c in prediabetic adults: a double-blind, randomized, placebo-controlled study. Nutrients. (2024) 16:2205. doi: 10.3390/nu16142205, 39064648 PMC11280236

[61] de Nies L Busi SB Tsenkova M Halder R Letellier E Wilmes P. Evolution of the murine gut resistome following broad-spectrum antibiotic treatment. Nat Commun. (2022) 13:2296. doi: 10.1038/s41467-022-29919-9, 35484157 PMC9051133

[62] Guerra I Ferreira HB Melo T Rocha H Moreira S Diogo L . Mitochondrial fatty acid β-oxidation disorders: from disease to lipidomic studies-a critical review. Int J Mol Sci. (2022) 23:13933. doi: 10.3390/ijms232213933, 36430419 PMC9696092

[63] Kunkemoeller B Prendeville H McIntyre C Temesgen A Loftus RM Yao C . The source of dietary fat influences anti-tumour immunity in obese mice. Nat Metab. (2025) 7:1630–45. doi: 10.1038/s42255-025-01330-w 40715760 PMC12373505

[64] Liu M Zheng X Sun C Zhou Q Liu B Xu P. Tea tree oil mediates antioxidant factors relish and Nrf2-autophagy axis regulating the lipid metabolism of *macrobrachium rosenbergii*. Antioxidants. (2022) 11:2260. doi: 10.3390/antiox11112260, 36421446 PMC9686997

[65] Xiang F Zhang Z Xie J Xiong S Yang C Liao D . Comprehensive review of the expanding roles of the carnitine pool in metabolic physiology: beyond fatty acid oxidation. J Transl Med. (2025) 23:324. doi: 10.1186/s12967-025-06341-5, 40087749 PMC11907856

[66] Wang M Ma LJ Yang Y Xiao Z Wan JB. N-3 polyunsaturated fatty acids for the management of alcoholic liver disease: a critical review. Crit Rev Food Sci Nutr. (2019) 59:S116–29. doi: 10.1080/10408398.2018.1544542, 30580553

[67] Yang X Li X Hu M Huang J Yu S Zeng H . EPA and DHA differentially improve insulin resistance by reducing adipose tissue inflammation-targeting GPR120/PPARγ pathway. J Nutr Biochem. (2024) 130:109648. doi: 10.1016/j.jnutbio.2024.109648 38631512

[68] Todisco S Santarsiero A Convertini P De Stefano G Gilio M Iacobazzi V . PPAR alpha as a metabolic modulator of the liver: role in the pathogenesis of nonalcoholic steatohepatitis (NASH). Biology. (2022) 11:792. doi: 10.3390/biology11050792, 35625520 PMC9138523

[69] Wang X Wang J Ying C Xing Y Su X Men K. Fenofibrate alleviates NAFLD by enhancing the PPARα/PGC-1α signaling pathway coupling mitochondrial function. BMC Pharmacol Toxicol. (2024) 25:7. doi: 10.1186/s40360-023-00730-6, 38173037 PMC10765888

[70] Mohamed AE Mahmoud AM Mohamed WR Mohamed T. Femtosecond laser attenuates oxidative stress, inflammation, and liver fibrosis in rats: possible role of PPARγ and Nrf2/HO-1 signaling. Life Sci. (2022) 307:120877. doi: 10.1016/j.lfs.2022.120877 35963297

[71] Grabacka M Płonka PM Pierzchalska M. The PPARα regulation of the gut physiology in regard to interaction with microbiota, intestinal immunity, metabolism, and permeability. Int J Mol Sci. (2022) 23:14156. doi: 10.3390/ijms232214156, 36430628 PMC9696208

[72] Zhang N Guan C Liu Z Li C Yang C Xu L . Calycosin attenuates renal ischemia/reperfusion injury by suppressing NF-κB mediated inflammation via PPARγ/EGR1 pathway. Front Pharmacol. (2022) 13:970616. doi: 10.3389/fphar.2022.970616, 36278223 PMC9585199

[73] Zamanian MY Alsaab HO Golmohammadi M Yumashev A Jabba AM Abid MK . NF-κB pathway as a molecular target for curcumin in diabetes mellitus treatment: focusing on oxidative stress and inflammation. Cell Biochem Funct. (2024) 42:e4030. doi: 10.1002/cbf.4030, 38720663

