# Supplementary material for: Ursodeoxycholic acid alleviates high-fat diet-induced liver injury by modulating gut microbiota-mediated bile acid metabolism: an integrated microbiota-metabolomics analysis
Source: Front Nutr. 2026 Jan 26;13:1714100. doi: 10.3389/fnut.2026.1714100 (PMC12883397; doi:10.3389/fnut.2026.1714100)
Supplement: Supplementary file 1 [file Table_1.docx]

Table S1. Primer sequences for quantitative PCR (qPCR)

| Genes | Forward primer (5′→3′) | Reverse primer (5′→3′) |
| --- | --- | --- |
| Mouse-CYP27A1 | GGACAACCTCCTTTGGGACTTACAC | TTGTGGTCTCGGTGGTCCTTCC |
| Mouse-NTCP | TTCACTGGGTCGGAGGATGGAG | CAGCCAAGCGAGAGCATGATGAG |
| Mouse-ASBT | CTGGTGCTTCTGTGGACTTGGC | CAGGAATCGCCTTCGCAGACAG |
| Mouse-BSEP | CTTCCATCCGTCAACGATCTAAGTCTC | GGTTCAACTTCTTCCACAAGCACATC |
| Mouse-OSTβ | TCAGGAGCAGAAACATGGACC | CTTAGGAAGACCTGGCTGTTGT |
| Mouse-Srebp-1 | CTGGTGAGTGGAGGGACCA | TGTGTGCACTTCGTAGGGTC |
| Mouse-CPT1a | CTGAGCCATGAAGCCCTCAA | ACGCCACTCACGATGTTCTT |
| Mouse-β-actin | GGCTGTATTCCCCTCCATCG | CCAGTTGGTAACAATGCCATGT |
| Human-CYP7A1 | CAAGGAATCGCTGAGGCTTTC | ACCGTCCTCAAGGTGCAAAGT |
| Human-CYP27A1 | CGGCAACGGAGCTTAGAGG | GGCATAGCCTTGAACGAACA |
| Human-β-actin | CATGTACGTTGCTATCCAGGC | CTCCTTAATGTCACGCACGAT |

Table S2. Serum differential metabolites regulated by UDCA treatment in NAFLD mouse model (ESI^+^ mode)

| **No.** | **Metabolite** | **Formula** | **HMDB** | **m/z** | **RT(min)** | **Which-max** |
| --- | --- | --- | --- | --- | --- | --- |
| 1 | Cinnamoylglycine | C11H11NO3 | HMDB0011621 | 206.08092 | 5.654 | ↑ |
| 2 | Hexanoylcarnitine | C13H25NO4 | HMDB0000756 | 260.18496 | 5.469 | ↓ |
| 3 | CAR 18:1 | C25H48NO4 | HMDB0005065 | 426.3566 | 8.083 | ↓ |
| 4 | CAR 18:0 | C25H50NO4 | HMDB0000848 | 428.37248 | 8.568 | ↓ |
| 5 | CAR 22:0 | C29H57NO4 | HMDB0062468 | 484.43625 | 9.726 | ↓ |
| 6 | CAR 20:1 | C27H52NO4 | -- | 454.3877 | 8.703 | ↓ |
| 7 | CAR 20:0 | C27H54NO4 | -- | 456.40302 | 9.16 | ↓ |
| 8 | Decanoylcarnitine | C17H33NO4 | HMDB0000651 | 316.24738 | 6.274 | ↓ |
| 9 | CAR 12:1 | C19H36NO4 | HMDB0002250 | 342.26267 | 6.483 | ↓ |
| 10 | PC O-19:4 | C27H48NO7P | -- | 530.3229 | 8.683 | ↓ |
| 11 | CAR 20:2 | C27H50NO4 | -- | 452.37192 | 8.277 | ↓ |
| 12 | CAR 17:0 | C24H48NO4 | -- | 414.35639 | 8.245 | ↓ |
| 13 | 4-Guanidinobutanoic acid | C5H11N3O2 | HMDB0003464 | 146.09198 | 5.269 | ↑ |
| 14 | 6-Deoxy-D-glucose | C6H12O5 | HMDB0247051 | 187.05734 | 1.392 | ↓ |
| 15 | 13-HPODE | C18H32O4 | HMDB0003871 | 335.21857 | 6.963 | ↓ |
| 16 | N-Acetylornithine | C7H14N2O3 | HMDB0240589 | 175.10741 | 1.41 | ↑ |
| 17 | Estriol | C18H24O3 | HMDB0000153 | 311.15902 | 5.015 | ↑ |
| 18 | Riboflavin | C17H20N4O6 | HMDB0000244 | 377.14404 | 5.328 | ↓ |
| 19 | Estrone | C18H22O2 | HMDB0000145 | 293.14786 | 8.044 | ↑ |
| 20 | 10-Nitrolinoleate | C18H31NO4 | HMDB0005049 | 348.21595 | 5.715 | ↓ |

Table S3. Serum differential metabolites regulated by UDCA treatment in NAFLD mouse model (ESI^-^ mode)

| No. | Metabolite | Formula | HMDB | m/z | RT(min) | which-max |
| --- | --- | --- | --- | --- | --- | --- |
| 1 | (+/-)12(13)-DiHOME | C18H34O4 | HMDB0010201 | 313.23735 | 7.137 | ↑ |
| 2 | 14,15-Dehydrocrepenynic acid | C18H28O2 | -- | 275.20059 | 8.816 | ↑ |
| 3 | 17α-Hydroxypregnenolone | C21H32O3 | HMDB0000363 | 331.22678 | 7.845 | ↑ |
| 4 | D-α-Tocopherol | C29H50O2 | HMDB0001893 | 475.37812 | 10.922 | ↓ |
| 5 | (±)19(20)-DiHDPA | C22H34O4 | HMDB0010214 | 361.23741 | 7.357 | ↑ |
| 6 | (±)10(11)-EpDPA | C22H32O3 | -- | 343.22668 | 8.348 | ↑ |
| 7 | Methyl linolenate | C19H32O2 | HMDB0254600 | 291.23221 | 7.792 | ↓ |
| 8 | 11-keto Testosterone (CRM) | C19H26O3 | HMDB0258073 | 347.1852 | 8.264 | ↑ |
| 9 | 11(Z),14(Z),17(Z)-Eicosatrienoic acid | C20H34O2 | HMDB0060039 | 305.24763 | 8.113 | ↓ |
| 10 | 13,14-dihydro-15-keto-tetranor Prostaglandin D2 | C16H26O5 | -- | 279.15897 | 6.847 | ↓ |
| 11 | Methyl arachidonate | C21H34O2 | HMDB0062594 | 317.24751 | 8.284 | ↓ |
| 12 | 1-Linoleoyl-Rac-Glycerol | C21H38O4 | HMDB0242115 | 353.26862 | 7.61 | ↑ |
| 13 | FAHFA 16:0/14:1 | C30H56O4 | -- | 479.40927 | 11.553 | ↓ |
| 14 | Docosapentaenoic acid | C22H34O2 | HMDB0246621 | 329.24717 | 9.857 | ↑ |
| 15 | 1,11-Undecanedicarboxylic acid | C13H24O4 | HMDB0002327 | 243.15932 | 6.699 | ↓ |
| 16 | LPC 20:5 | C28H48NO7P | HMDB0010397 | 586.31311 | 8.623 | ↑ |
| 17 | Eicosapentaenoic acid | C20H30O2 | HMDB0001999 | 301.21589 | 9.176 | ↑ |
